# Optimized periphery-core interface increases fitness of the *Bacillus subtilis glmS* ribozyme

**DOI:** 10.1093/nar/gkae830

**Published:** 2024-09-25

**Authors:** Li-Eng D Yu, Elise N White, Sarah A Woodson

**Affiliations:** Program in Cell, Molecular and Developmental Biology and Biophysics, Johns Hopkins University, Baltimore, MD 21218, USA; Program in Molecular Biophysics, Johns Hopkins University, Baltimore, MD 21218, USA; T.C. Jenkins Department of Biophysics, Johns Hopkins University, Baltimore, MD 21218, USA

## Abstract

Like other functional RNAs, ribozymes encode a conserved catalytic center supported by peripheral domains that vary among ribozyme sub-families. To understand how core-periphery interactions contribute to ribozyme fitness, we compared the cleavage kinetics of all single base substitutions at 152 sites across the *Bacillus subtilis glmS* ribozyme by high-throughput sequencing (*k*-seq). The *in vitro* activity map mirrored phylogenetic sequence conservation in *glmS* ribozymes, indicating that biological fitness reports all biochemically important positions. The *k*-seq results and folding assays showed that most deleterious mutations lower activity by impairing ribozyme self-assembly. All-atom molecular dynamics simulations of the complete ribozyme revealed how individual mutations in the core or the IL4 peripheral loop introduce a non-native tertiary interface that rewires the catalytic center, eliminating activity. We conclude that the need to avoid non-native helix packing powerfully constrains the evolution of tertiary structure motifs in RNA.

## Graphical Abstract

**Graphical Abstract:**
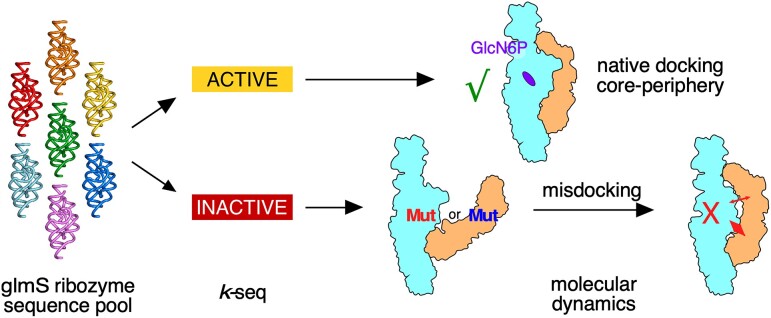

## Introduction

Ribozymes fold into intricate tertiary structures that create active sites for catalysis or ligand recognition (1,2). In addition to a conserved core domain containing the active site, ribozymes frequently encode peripheral helices that form long-range tertiary interactions that stabilize the core (3–6). Cooperative assembly of peripheral tertiary interactions in ribozymes can reduce misfolding by favoring correct orientation of the RNA helices (7,8), increase thermostability of the RNA (9), and improve catalysis by fine-tuning the conformation of the active site (10). Because the peripheral helices act as independent modules, their structures may change or expand during the evolution of RNA subclasses, although their stabilizing function is preserved (11,12).

The constraints on the molecular interactions needed for catalysis or ligand binding conserve active sites within RNA families (12–14). For example, a comparison of small hydrolytic ribozymes suggested that these RNAs use nearly identical interactions to catalyze self-cleavage, although the scaffold that achieves these interactions is different in each ribozyme family (15,16). By contrast, the selective pressures that maintain the peripheral interactions in ribozymes and riboswitch families are less understood. Although peripheral domains are less conserved overall, mutations that disrupt peripheral tertiary interaction motifs can be deleterious, especially under suboptimal conditions (17–20). This sensitivity to mutation outside the active site suggests that the assembly of native structures in RNA constrains fitness landscapes, which are rugged (21,22).

To understand how core and peripheral interactions each contribute to the fitness of functional RNAs, we performed a large mutational screen of the *Bacillus subtilis* (*Bsu*) *glmS* ribozyme. The *glmS* ribozyme down-regulates expression of glucosamine synthase (GlmS) in response to rising levels of glucosamine-6-phosphate (GlcN6P), a precursor for cell wall biosynthesis (23). Binding of GlcN6P to the folded ribozyme in the *glmS* 5′ UTR triggers cleavage and turnover of *glmS* mRNA (23,24). Rather than changing the ribozyme structure (25,26), GlcN6P activates the ribozyme by participating in acid/base catalysis of phosphodiester cleavage (27–29).

**Figure 1.**
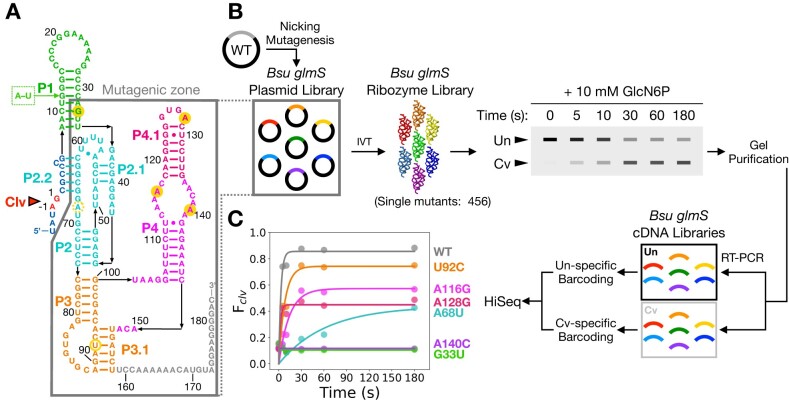
Measuring the activity of *glmS* ribozyme variants by k-seq. (**A**) The secondary structure of the *Bacillus subtilis glmS* ribozyme (modified from (23)) with paired (P) regions colored by domain. All possible (456) single mutations in the 152 nt mutagenic zone (gray outline) were scored by a variation on *k*-seq (38). (**B**) Workflow overview. The library of ribozyme variants (RzLby) was allowed to self-cleave in the presence of 10 mM Mg^2+^ and 10 mM GlcN6P, purified by PAGE and reverse transcribed into 12 barcoded output cDNA sub-libraries that were pooled and sequenced. See Methods and Supplementary Figures S1-S3 for details. (**C**) Self-cleavage of representative variants (gold circles in panel A) measured by *k*-seq. The WT ribozyme displayed superior activity with 86 ± 5% yield at 180 s. See Supplementary Table S2 for cleavage rates and Supplementary Figure S4 for comparison with PAGE results.

Sequence alignments and chemical probing data showed that the *glmS* ribozyme is comprised of a conserved core domain (paired regions P1–P2) containing the active site for GlcN6P-dependent cleavage (30), and a downstream peripheral P3/P4 domain that increases the stability of the core domain (23,24,31). Crystallographic structures of *glmS* ribozymes from *Thermoanaerobacter tengcongensis* (*Tte*) and *Bacillus anthracis* revealed a core double pseudoknot (P2-2.1–2.2) stacked with the adjacent P1 and P3 helices (25,32) (Figure 1A). An internal loop in P4 (IL4) packs against the core, stabilizing the conformation of the P2.1 helix via an extended purine stack (25). This helix interface is pinned in place via the P3/P3.1 pseudoknot and the L4 tetraloop-P1 helical receptor interaction.

In vitro selection of *B. cereus glmS* ribozyme variants previously showed that residues in the core and the peripheral IL4 loop that preserve the double pseudoknot are important for activity (33), as expected based on the phylogenetic conservation of these elements. By contrast, the requirement for GlcN6P activation was obviated by only a few mutations in the active site itself (34). A fitness landscape of the 54-nt core of *Tte glmS* ribozyme was obtained using a high-throughput assay for self-cleavage as a function of cofactor concentration (35). The strong correlation between sequence conservation and catalytic rate suggested that catalytic efficiency rather than ligand binding drives phylogenetic conservation of the ribozyme core (35).

Most efforts to map ribozyme fitness landscapes have focused on small ribozymes or a limited number of positions within a larger RNA, for which the sequence space can be fully covered (21,22,35,36). However, this approach is less suited to assessing how long-range tertiary contacts contribute to the function of large ribozymes. Here, *in vitro* self-cleavage was coupled with high-throughput sequencing to measure the activity of all possible 456 single base substitutions at 152 positions of the *Bsu glmS* ribozyme covering both the catalytic core and peripheral P3/P4 domain (Figure 1). All-atom molecular dynamics (MD) simulations of select variants were performed to probe the sources of cooperativity in this large RNA. The combined results illuminate how critical tertiary interactions improve fitness by enhancing the stability of the native core while suppressing tertiary interactions that trap the core in an inactive conformation. The results suggest that the evolution of functional RNA sequences is powerfully constrained by the need to avoid competing non-native structures.

## Materials and methods

### Reagents

Standard reagents were used including QIAprep Spin Miniprep kit, (Qiagen, #27104), Monarch PCR & DNA Cleanup (NEB), *ExoI* (NEB #M0293S), SuperScript IV One-Step RT-PCR System kit (Invitrogen, #12594025). Custom primers and oligonucleotides were obtained from IDT with standard desalting purification (Supplementary Table S1).

### Biological resources

The *glmS* ribozyme sequence (23) was amplified from *B. subtilis* strain UD1022 locus CP011534 (nucleotide 501–670). pBlueScript II SK(−)-*BbvcI*-*trans*-*glmSCC* containing the base substitution U31C in helix P1 was constructed as described in Supplemental Methods and propagated in *Escherichia coli* DH5α (NEB, C2987H).

### Library preparation and *k*-seq

Plasmid pBlueScript II SK(−)-*BbvcI*-*trans*-*glmSCC* encoding the parental *B. subtilis glmS* ribozyme was used to generate a library of ribozyme variants by nicking mutagenesis (37), covering 152 positions from A32 to C183 (see Supplemental Methods for details). Pooled DNA was extended by nested PCR to add a T7 promoter and 18 nt upstream of the ribozyme cleavage site, and the templates transcribed *in vitro* to yield an input library of 276-nt uncleaved RNA. The *cis-glmS*CC ribozyme pool additionally contains the base pair substitution A13G,U31C to stabilize P1. The activity of each variant was estimated using a variation of *k*-seq, which was first developed to measure the activity of small ribozymes (38). For each reaction timepoint, 50 pmol ribozyme library was renatured in 50 mM K-HEPES pH 7, 10 mM MgCl_2_, 50 mM NaCl, 5 mM KCl using a multi-step protocol (29), then allowed to self-cleave for 0, 5, 10, 30, 60, or 180 s at 37°C in 10 mM GlcN6P (pH 6.5–6.8). Reactions were quenched with an equal volume 100 mM EDTA and the cleaved and un-cleaved ribozyme separated by 8 M urea 5% PAGE to generate a total of 12 RNA pools. Barcoded Illumina sequencing libraries were prepared from each pool and sequenced by 150-bp paired-end HiSeq (Novogene). The cleaved at each reaction time, *F*_clv_(*t*), was calculated for each variant (39) from aligned reads in cleaved and uncleaved pools, after quality screening and spike-in normalization (Supplemental Methods; Supplementary Figures S1–S3). *F*_clv_(*t*) versus *t* was fit by exponential rate equations to obtain the observed cleavage rate and product yield at 180 s for every variant (Supplementary Figure S4).

### Self-cleavage and folding assays

The observed self-cleavage rate was measured for individual variants as described above with 5 pmol RNA per reaction. Following 5% PAGE, gels were stained with SYBR-Gold and scanned (Amersham Typhoon 5 Biomolecular Imager). For the cleavage assays with anti-IL4 RNA oligomers, 0.5 pmol ribozyme was premixed with both oligomers (200 pmol each) before renaturation. For cleavage at varying temperatures, 2 pmol ribozyme was pre-folded as above, then incubated at the desired temperature for ≥3 min before addition of 10 mM GlcN6P (final). For native PAGE folding assays, refolded RNA was briefly mixed with 10% glycerol and immediately loaded on native 8% PAGE in 66 mM HEPES, 34 mM Tris, 0.1 mM EDTA and 3 mM MgCl_2_ (40).

### All-atom molecular dynamics simulations

A homology model of the *Bsu* ribozyme was templated from PDB 2H0Z (25) as described in Supplemental Methods and Supplementary Figure S5. To simulate folding of the ribozyme prior to self-cleavage, the GlcN6P co-factor was omitted from the model. All-atom MD simulations were performed with GROMACS 2019.4 (41) through shared computing resources (42). RNA was represented by Amber99 force field (43) with Chen-Garcia (44) and Steinbrecher-Case modifications (45). Models were solvated with TIP4P-Ew, 150 mM KCl and 10 mM MgCl_2_ with tuned Mg^2+^ parameters (46). Following equilibration, the WT, A114C and A40G ribozyme models were simulated at 310 K for roughly 50 ns across 10 replicas, for a total of 500 ns of sampling per model. See the Supplemental Methods for further details of the simulation procedures.

### Statistical analyses

A full description of sequencing data analysis, modeling and error analysis is provided in the Supplemental Methods. Spike-in control RNAs were used to evaluate sequencing accuracy and mutation calling errors. Uncertainties of fitted parameters were taken from the variance of the fit for large data sets or standard error of the mean among independent trials for individual biochemical assays. The sample size and numbers of replicates for each experiment are indicated in the figure legends and tables.

### Data availability/sequence data

Sequencing data have been deposited in NCBI’s Gene Expression Omnibus (47) and are accessible through GEO Series accession number GSE261357.

### Data availability/novel programs, software, algorithms

MD trajectories and custom scripts used for data processing and analysis are included with the publicly available Johns Hopkins Data Services repository associated with this paper.

## Results

### *k*-seq measures the activity of *glmS* ribozyme variants

To determine a fitness landscape for folding of the *Bsu glmS* ribozyme, we developed a protocol in which the extent of self-cleavage was measured by high-throughput sequencing (Figure 1B). Our protocol is related to *k*-seq, in which sequencing reacted and unreacted RNA pools at various reaction times allows the reaction kinetics of many variants to be reconstructed (38). The parental precursor RNA, hereafter named *cis-glmSCC*, included 19 *glmS* nucleotides upstream of the ribozyme cleavage site to avoid contributions from intermolecular substrate binding (48). The P1 helix was also stabilized by exchanging A12-U31 for a G-C base pair (Figure 1A).

To ensure that the rate of self-cleavage reflects the competence of a given variant to form the native structure, we used 10 mM MgCl_2_ and saturating (10 mM) GlcN6P cofactor. Under these assay conditions, 92% ± 2% *cis-glmSCC* ribozyme was cleaved with *k*_obs_ = 0.20 ± 0.02 s^−1^ (Supplementary Figure S1; Supplementary Table S2), comparable to the cleavage kinetics of *B. anthracis glmS* ribozyme (29). Self-cleavage in saturating GlcN6P is much faster than Mg^2+^-dependent folding of the *glmS* ribozyme (49). Thus, under the conditions of our experiments, the cleavage kinetics is expected to report on proper folding of the ribozyme.

A plasmid library encoding all possible single base substitutions in a 152 bp window covering the P1 helix, ribozyme core, P3 pseudoknot junction, peripheral P4 region, and the 3′-tail (Figure 1A, circled area) was generated by nicking mutagenesis (37). After *in vitro* transcription, renaturation at 10 mM Mg^2+^, and the addition of 10 mM GlcN6P, cleaved and uncleaved RNA pools at six reaction times (0–180 s) were isolated by PAGE (Figure 1B) and the 152 nt region was sequenced using paired-end high-throughput sequencing. The activity of each variant was determined from the number of reads in the cleaved and uncleaved libraries over the reaction time course, after normalization to spike-in control RNAs (Supplementary Figure S2-S3). We were able to estimate the endpoint and rate of self-cleavage for all 456 single base substitutions (Figure 1A).

The input ribozyme pool contained many variants deficient in self-cleavage, as judged by its lower average cleavage rate (0.06 ± 0.01 s^−1^) and 24% lower yield relative to the parental *cis-glmSCC* ribozyme (Supplementary Figure S1). To assess whether *k*-seq accurately measures the activity of individual variants, we measured the ribozyme cleavage kinetics of several variants by conventional PAGE. The results of the two assays were highly similar (*P* < 0.001; Supplementary Figure S4), although *k*-seq overestimated the product yield in a few instances owing to high background at zero time. Random sequencing errors that lead to false mutation calling were judged to over-estimate self-cleavage of individual ribozyme variants by ≤2% (see Supplementary Methods). Because the RNA pool was sequenced at only six reaction times, we obtained only upper or lower bound estimates of reaction rates for variants that self-cleave very rapidly or to a low extent.

Complete coverage of single variants produced an informative sketch of the *glmS* ribozyme fitness landscape that correlated well with the known functions of the *glmS* ribozyme domains. Mutations at residues near the co-factor binding site reduced the reaction rate (e.g. A68U in Figure 1C; Supplementary Table S2), consistent with impaired catalysis. By contrast, mutations at residues that participate in long-range tertiary interactions, such as the L4-P1 GNRA tetraloop-receptor motif and the IL4-P2.1 interface, reduced the yield of cleaved product without appreciably changing the initial reaction rate (A116G, A128G, A140C in Figure 1C). This pattern was consistent with partitioning of the RNA population into misfolded structures. The low extent of reaction is unlikely to arise from a shift in the equilibrium between cleavage and ligation, as product release is favorable and *glmS* ribozymes have not been reported to catalyze self-ligation (50).

### Activity landscape of core and peripheral helices

**Figure 2.**
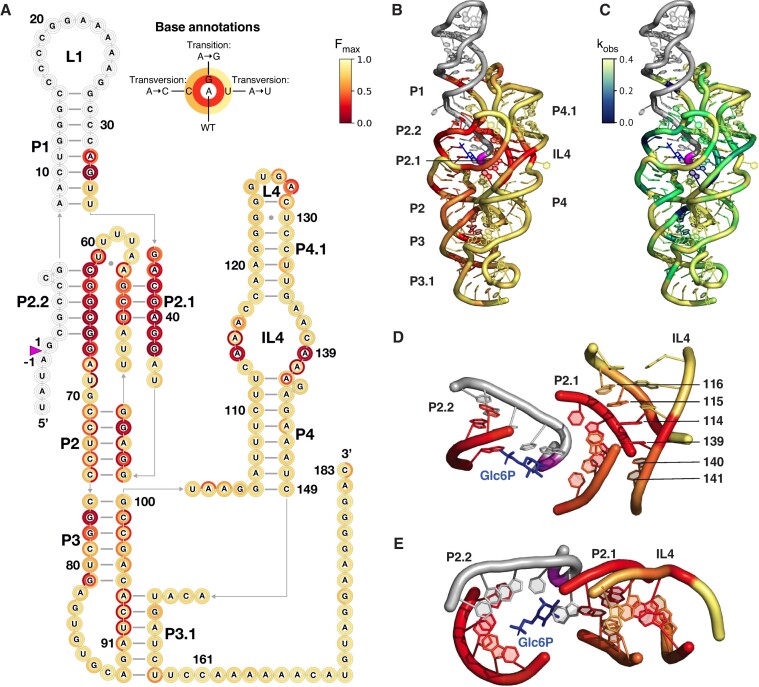
Self-cleavage fitness landscape of the *B. subtilis glmS* ribozyme. (**A**) Heatmap of *k*-seq reaction endpoints for all 456 single mutations. At each residue, the letter indicates the WT base, the inner ring shows the activity of the transition mutation and the two halves of the outer ring represent the transversions in alphabetical order. Paired (P) regions are labeled. See Supplementary Figure S6 for observed cleavage rates and Dataset 1 for parameters. (**B**) Ribozyme fitness projected on *Bsu* 3D homology model (Supplementary Figure S5) templated from PDB 2h0z (25). Colors represent the average activity of three base substitution variants at each position. (**C**) Fitness measured by observed reaction rates, capped at a maximum of 0.4 s^−1^ and minimum of 0.01 s^−1^. (**D, E**) Close-up of core-IL4 interaction indicating deleterious mutations at the helix interfaces. Blue sticks, cofactor analog glucose-6-phosphate. (Views adapted from (25).)

We mapped the relative activities of all 456 single base substitutions onto the *Bsu* ribozyme secondary structure (Figure 2A). We also mapped them onto a 3D model of the *Bsu* ribozyme (Figure 2B) templated from the crystallographic structure of the *Tte* ribozyme (25). As expected, base substitutions at positions known to be functionally important reduced ribozyme activity (Figure 2A, B). In the ribozyme core (G36 to C75), 19 out of 40 sites were susceptible to mutation. At these positions, at least two of the three base substitutions reduced the yield of cleaved product to less than 30% (red, Figure 2A). Core helices P2, P2.1 and P2.2 were more vulnerable to mutation than single-stranded segments joining the core helices (Supplementary Figure S6), in agreement with a previous fitness landscape of the *Tte* ribozyme core (35). Mutations in core helices typically reduced the population of active RNA. Thus, base mismatches in this region likely reduce activity by compromising the double-pseudoknot (51) that creates the active site for GlcN6P binding. Conversely, base substitutions in loops surrounding the GlcN6P pocket and the active site tended to reduce the rate of reaction (Figure 2C, Supplementary Figure S6), in some cases without reducing yield.

In contrast with core residues, the P3 pseudoknot junction (24/31 sites) and the peripheral P4 domain (46/52 sites) tolerated single base substitutions (Figure 2A), consistent with previous *in vitro* selection results (33). The few detrimental mutations in P3 and P4 clustered at conserved positions (30) that are crucial for the ribozyme tertiary structure. In some of these sites, transitions (pyrimidine to pyrimidine or purine to purine) were tolerated, whereas transversions (pyrimidine to purine or purine to pyrimidine) were not. For example, replacement of U92 in the P3.1 helix with C had little impact (Figure 1C; Figure 2A), but replacement with A or G reduced activity to ∼20% (Figure 2A). This pattern suggests the importance of helix packing. For example, mutations in conserved G-C base pairs in P3 that form a base triple with the adjacent J3/4 linker (25) were detrimental (Figure 2A), as expected. Similarly, deleterious mutations in P3.1 resided in the strand facing the L3 loop (G89 to A93).

The L4 tetraloop and its G-C receptor in P1 were sensitive to mutation, confirming the importance of this long-range tertiary interaction for maintaining the activity of the full-length ribozyme. The docking site in P1, G33, was intolerant of any base substitution, as was its partner A128 in L4 (Figure 2A, B). This pattern is consistent with the docking geometry, which is identical to that of the L2 tetraloop-P8 receptor in the phage T4 *td* ribozyme (52). By contrast, mutations at G125 in L4 were harmless. This agrees with the importance of G125 for maintaining the conformation of the GNRA tetraloop but not interacting with the receptor, as seen in structures of RNase P and P4-P6 RNA (53,54).

More strikingly, we observed that consecutive adenines in the internal loop IL4 (A114-116 and A139-141) were particularly vulnerable to mutation (Figure 2A). For example, variant A116G reduced the yield to 57 ± 6% and lowered the cleavage rate to 0.07 ± 0.03 s^−1^, which is 20% the WT value (0.36 ± 0.14 s^−1^) (Supplementary Table S2). This pattern differed from other peripheral mutations that reduce yield but not cleavage rate. In the crystal structures of the *Tte* and *B. anthracis glmS* ribozymes, the IL4 purines stack in the minor grove of P2.1 where they establish an extensive set of base triples that reinforce the ligand binding pocket (Figure 2D, E) (25,32). A114 and A139 at the center of the purine stack were more sensitive to substitution than A116 and A141 at the edges of the stack. From these results, we speculated that IL4 variants may cause a rearrangement of the core-IL4 interface that leads to improper organization of the active site.

### IL4 mutations change the ribozyme folding pathway

**Figure 3.**
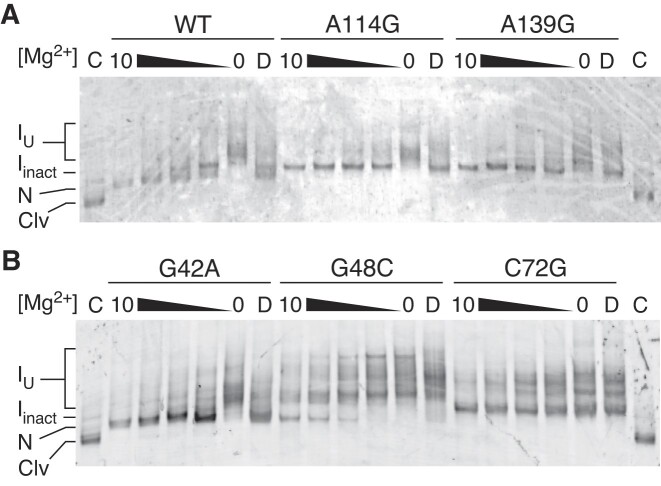
IL4 mutations favor an inactive conformation. (**A**) Mg^2+^-dependent folding of the WT ribozyme and IL4 variants at 0, 1.25, 2.5, 5, 10 mM Mg^2+^ by native 8% PAGE. C, control partially cleaved WT control; D, denatured RNA in formamide. Clv, cleaved ribozyme; N, native uncleaved ribozyme; *I*_inact_, compact structure with misdocked IL4; *I*_U_, less compact, inactive structures. (**B**) Folding of inactive core mutants, labeled as in (A). G42A disrupts the active site; G48C and C72G introduce a mismatch in P2. Results of one trial are shown.

Mutations that weaken long-range tertiary interactions or core pseudoknots may make the ribozyme more likely to misfold into an inactive structure (7,20). To investigate whether such mutations perturb the folding pathway of the *Bsu glmS* ribozyme, we refolded the parental ‘WT’ RNA and selected variants at different Mg^2+^ concentrations and analyzed the conformers by native PAGE (Figure 3). At 10 mM Mg^2+^, ∼90% of the WT ribozyme folded into a compact structure (lowest band, N; Figure 3A) that we assigned to the native state because 92% ± 2% of the ribozyme self-cleaved under these conditions (Supplementary Figure S1). About 10% of the RNA formed a non-native structure that migrated more slowly in the gel (*I*_inact_). At low Mg^2+^, the ribozyme formed uncompact structures (*I*_U_) that converted to native RNA with increasing Mg^2+^ concentration.

Unlike the WT ribozyme, inactive IL4 variants (A114G and A139G) formed the non-native structure at all Mg^2+^ concentrations tested (Figure 3A). This result showed that the IL4 mutations do not cause the RNA to unfold. Instead, these IL4 mutations favor an alternative conformation and suppress formation of the native structure. The active site mutant G42A in P2.1 formed the native conformation (Figure 3B), in agreement with the finding that the homologous G40A mutation in *Tte glmS* ribozyme eliminated catalysis without perturbing its structure (27). By contrast, mutations G48C and C72G in P2 dramatically destabilized the ribozyme, leading to multiple misfolded conformers that traveled more slowly in the native gel (Figure 3B). Since G48–C72 co-vary (30), complementarity at this position within the core is likely important for maintaining the overall structure of the core. These results supported our hypothesis that many deleterious mutations reduce fitness by compromising self-assembly rather than undermining catalysis.

### IL4 mutations create an inactive metastable core

**Figure 4.**
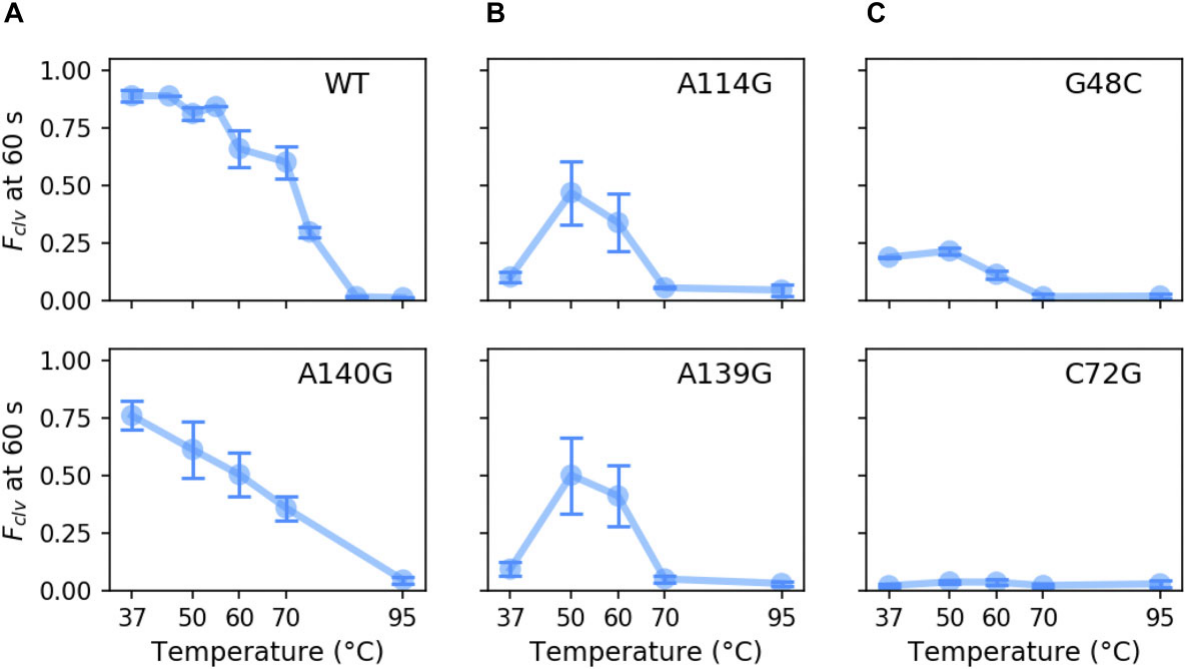
Misfolded IL4 is metastable. (**A**) Fraction cleaved at 60 s for the WT and partially active IL4 variant A140G at varying temperatures. (**B**) IL4 variants with little or no activity at 37°C regain activity at 50°C. (**C**) Core mutations reduce activity at all temperatures tested. Symbols, mean and S.D. for 3–4 trials. See Supplementary Figure S7-S8 for further data.

In earlier studies (23,31,55), the peripheral P4 domain was dispensible for self-cleavage, raising the question why IL4 mutations favor an inactive form of the ribozyme. To investigate how IL4 mutations destabilize the native structure, we expressed 18 variants carrying a single mutation in the purine stack (nt. 114–116 and nt. 139–141) and measured the extent of self-cleavage from 37 to 95°C (Figure 4 and Supplementary Figure S7). The parental WT ribozyme was active up to 70°C, but was inactive at 90°C. The A140G mutant was unstable, gradually losing activity between 50 and 95°C. Strikingly, A114 or A139 variants were completely inactive at 37°C yet regained activity at 50–60°C, before losing activity again at 70°C (Figure 4B). This pattern was observed for most variants at these positions 114 and 139, which lie at the center of the IL4 purine stack (Supplementary Figure S7). This non-linear temperature dependence suggested that mutations in IL4 favor a non-native structure at 37°C that converts to an active structure at higher temperatures (Supplementary Figure S8; Supplementary Table S3). Unlike IL4 variants, variants in the ribozyme core that were inactive at 37°C remained inactive at all temperatures tested (Figure 4C).

**Figure 5.**
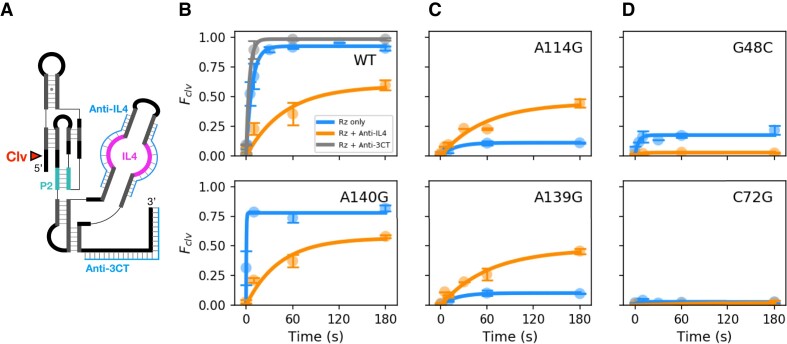
Misdocking of IL4 drives misfolding of the ribozyme core. (**A**) Antisense oligonucleotides block interactions between the core and P4 domain (anti-IL4); control base pairs with downstream extension (anti-3CT). Native gel shift experiments showed that anti-3CT hybridizes with WT ribozyme RNA (Supplementary Figure S9). (**B**) Self-cleavage of WT and active A140G mutant at 37°C, RNA only (blue), RNA + Anti-IL4 (orange) or RNA + Anti-3CT (gray). Blocking IL4 docking lowers the endpoint at 180 s by 33% and the cleavage rate 3.4-fold (0.07 ± 0.03 s^−1^) compared to the absence of anti-IL4. Symbols indicate the mean ± S.E.M. of 3 independent trials (7 for WT Rz). (**C**) Self-cleavage of inactive IL4 variants as in panel B. Activity increases when IL4 docking is blocked. (**D**) Inactive P2 variants are not rescued by blocking IL4. See Supplementary Table S4 for parameters.

To test whether the low activity of IL4 variants at 37°C was due to an aberrant interface between IL4 and the core, we measured the cleavage kinetics in the presence of two anti-sense oligonucleotides (anti-IL4) that bind IL4 and disrupt its interaction with the core (Figure 5A). As expected, the anti-IL4 oligomers reduced the activity of the WT ribozyme by ∼33%, whereas an anti-sense oligonucleotide complementary to the 3′ tail (anti-3CT) had no effect (Figure 5B; Supplementary Table S4). A similar result was obtained for the active A140G variant (Figure 5B). Thus, blocking the interaction between functional IL4 and the catalytic core reduced activity, confirming that IL4 normally contributes to ribozyme fitness by stabilizing the core in its native state.

Conversely, the anti-IL4 oligomers enhanced self-cleavage of inactive IL4 variants (A114G and A139G) from ∼10% to ∼50% (Figure 5C; Supplementary Table S4), raising the variants to the level of the WT ribozyme plus anti-IL4 (mean = 0.54, SD = 0.06; Figure 5B, C; Supplementary Table S4). This result suggested that IL4 mutations create a non-native tertiary interaction with P2.1 that is relieved if interactions between IL4 and P2.1 are blocked. From this result, we concluded that improper IL4 interactions must sequester the ribozyme core in an inactive state. Altogether, these results demonstrated that the sequence of IL4 is not only optimized to stabilize the native P2.1 helix, but to also avoid non-native helix packing that inactivates the core.

### All-atom simulations reveal perturbations to ribozyme core

**Figure 6.**
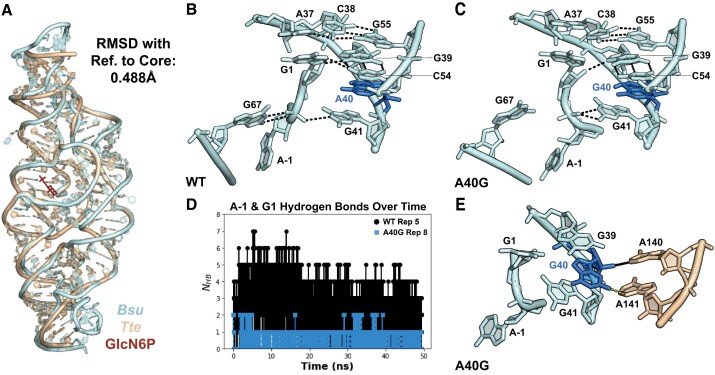
Core mutation rewires H-bonding network in the active site. (**A**) Initial homology model of the *Bsu* ribozyme (pale cyan) compared to the crystal structure of the *Tte* ribozyme (wheat) complexed with the GlcN6P cofactor (firebrick). (**B**) Representative snapshot of the WT *Bsu* ribozyme core (replica 5) illustrates how core residues A40, G41, C54 and G67 position the cleavage site between residues A-1 and G1. (**C**) Snapshot of perturbed core of the A40G *Bsu* ribozyme (replica 8). The scissile phosphate of G1 has reoriented due to loss of contacts to G67, G40 and C54. Contacts between A37 and C38 are also disrupted. (**D**) Number of hydrogen bonds involving A-1, G1, G41, G67 and C54 from WT replica 5 and A40G replica 8 shows depletion of native contacts in A40G. Based on the *Tte* crystal structure, 5 hydrogen bonds are expected to form among these residues. (**E**) G40 N2 forms a non-native H-bond with A141 N3 (yellow) in IL4 (wheat) in 7/10 of A40G replicas. See Supplementary Table S6 for hydrogen bond occupancy across all 10 replicas and Supplementary Table S7 for weak restraints applied to RNA during the simulation.

To understand how base substitutions in the catalytic core and in IL4 favor inactive structures, we performed all-atom molecular dynamics (MD) simulations on the complete *Bsu glmS* ribozyme (nts –1 to 159) starting from our homology model. The final equilibrated WT *Bsu* model retained all the homologous base pairs and tertiary interactions in the *Tte* ribozyme template (Figure 6A). Although GlcN6P binding helps position the reactive phosphodiester for in-line attack (32,56,57), it is not required for preorganization of active site or overall folding of the ribozyme (25,26). As we were interested in self-assembly prior to cleavage, GlcN6P was not included in the simulations. We next individually introduced two deleterious mutations, A40G in the ribozyme core and A114C in IL4. A114C has a similar effect on activity as A114G but may perturb stacking interactions in IL4 more strongly. To observe how the ribozyme structure changes in response to these mutations, we simulated each model at 310 K for ∼50 ns across 10 replicas, for a total of 500 ns of sampling per model (Supplementary Table S5). This simulation timescale successfully captured large conformational rearrangements in other riboswitches (58,59). We found that this strategy was sufficient to observe rewiring of hydrogen bonding networks and base stacking interactions in the *Bsu glmS* ribozyme, explaining why these mutations reduce activity. These local perturbations may lead to further unfolding of the catalytic core over longer timescales, as observed biochemically (Figure 3).

*A40G*. A40 is part of an intricate network that connects conserved core residues with A-1 and G1 flanking the cleavage site (Figure 6B). This network was disrupted when A40 was replaced with G. In the native conformation, G39, G41, C54 and G67 contact the backbone of A-1 and G1, positioning the scissile phosphate for in-line attack by GlcN6P (56). Hydrogen bonds among these residues were visible in 80–94% of frames of WT replicas (Supplementary Table S6) but disrupted in 3/10 A40G replicas, allowing the scissile phosphate to adopt a cleavage incompetent state that was never observed in WT trajectories (Figure 6C,D). Hydrogen bonds between A-1 and G67 were missing in 97–99% of frames of perturbed A40G replicas. Additionally, a hydrogen bond from G1 O2′ to G39 O6 was also missing in nearly all frames. In one A40G replica, reorientation of the scissile phosphate was accompanied by disruption of the conserved A37*(C38–G55) base triple that connects P2.1 to P2.2 (Figure 6C, Movies S1 and S2). Additionally, the neighboring A56–U61 base pair was replaced by a non-native hydrogen bond between A37 and A56 in 64% of frames (Supplementary Figure S10).

A comparison of the WT and A40G models explains how a single base substitution perturbs so many interactions surrounding the scissile phosphate. Surprisingly, it introduces a new hydrogen bond from G40 N2 to A141 N3 in IL4 that cannot be made by A40 (Figure 6E). This non-native contact, present in 7/10 A40G replicas, pulls G40 towards IL4, ablating interactions between G40 and P2.1 in the core. Thus, the A40G mutation is deleterious because it promotes improper docking between the core and the peripheral domain.

**Figure 7.**
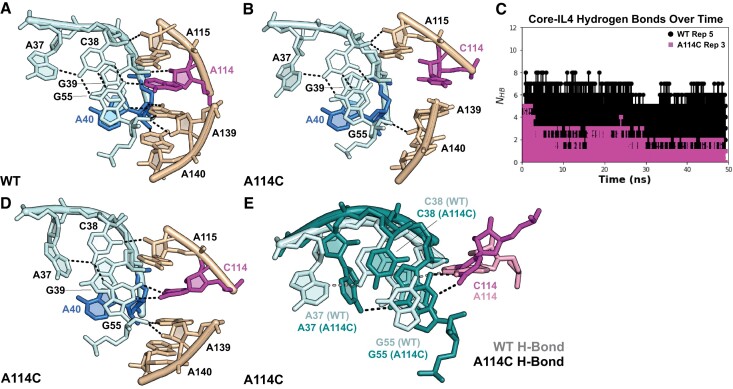
Misdocking of A114C IL4 induces misfolding of catalytic center. (**A**) Snapshot of the WT P2.1-IL4 interface (replica 10). A114 N1 hydrogen bonds with G55 N2, stabilizing the native architecture of P2.1. (**B**) Snapshot of A114C IL4 (replica 3) illustrating movement away from P2.1 observed in 7/10 A114C replicas. (**C**) Number of hydrogen bonds between core residues 38–40, 55 and IL4 residues 114, 139, 140 illustrating the loss of core-IL4 contacts in A114C. Six hydrogen bonds are expected to form between these residues. (**D**) Snapshot of misdocked A114C P2.1-IL4 interface (replica 9), which is representative of 2/10 A114C replicas. C114 forms a non-native hydrogen bond with G55, disrupting the conserved A37*(C38-G55) triple involved in GlcN6P recognition. (**E**) Superposition of the core-IL4 interface from WT replica 5 and A114C replica 5. In A114C, a non-native hydrogen bond between C114 and G55 unstacks G55 from P2.1. A37 flips out of plane, and a non-native hydrogen bond forms between A37 N6 and G39 O6.

*A114C*. A114 is part of the conserved IL4 purine stack that docks with the minor groove of P2.1, where A114 N1 hydrogen bonds with G55 N2 in P2.1, reinforcing the conserved A37*(C38-G55) triple (Figure 7A). This and other core-IL4 interactions were present in 96% of all frames in 9/10 WT replicas (Supplementary Table S6). In 7/10 A114C replicas, however, the center of the IL4 purine stack (C114, A139 and A140) moved away from the core, reducing core-IL4 contacts to 62–80% of frames (Supplementary Table S6). This movement was accompanied by reduced stacking in IL4 (58% A114C frames, compared to 91% WT frames). Undocking can firstly be explained by the pyrimidine substitution that puts C114 N3 out of hydrogen bonding distance to G55 N2 (7Å) compared with A114 N1 (3Å) (Figure 7B). Secondly, the ribose zipper between C114 O2′ and G39 O2′ is lost. Finally, A139 must swivel away from P2.1 by 130° to stack underneath C114 (Supplementary Figure S11).

Importantly, we observed aberrant IL4–P2.1 docking that restructured the core itself in two of the remaining three A114C replicas. In these trajectories, non-native hydrogen bonding between C114 N4 and G55 N3 pulled G55 out of P2.1, breaking the C38–G55 base pair and the ribose zipper between C114 O2′ and G39 O2′. In replica 9, the conserved A37*(C38–G55) triple was disrupted for 80% of the trajectory (Figure 7C, Movie S3 and S4). In replica 5, A37 flipped out of plane and formed a non-native contact with G39 in 9% of frames (A37 N6 to G39 O6; Figure 7E). Native contacts between the G39-C54 base pair and G1 that help position the scissile phosphate were absent in all frames of replicas 5 and 9. Therefore, the simulations reveal how misdocking with IL4 dislocates key residues in P2.1, rationalizing why A114 mutations trap the ribozyme core in inactive conformations.

## Discussion

Functional RNAs, such as ribozymes, are defined by conserved catalytic centers (reviewed in (60–62)) that are typically unstable without reinforcement by peripheral domains (63). Because the peripheral domains vary among ribozyme family members, their contribution to the fitness of a given sequence may be harder to discern. Our survey of single base substitutions in the *Bsu glmS* ribozyme shows that conserved residues throughout the ribozyme are needed for robust self-assembly (Figure 2). The self-cleavage activity map is consistent with phylogenetic sequence conservation (30,48), *in vitro* selection of active variants (33), and an activity map of the catalytic core (35). The agreement between sequence conservation and activity is strongest within the catalytic core, as expected, yet encompasses key peripheral tertiary interactions (Supplementary Figure S12). Information on the cleavage kinetics for variants in the core and peripheral domains provides a clearer view of constraints on ribozyme fitness and evolution. This view is elaborated by the all-atom MD simulations, which agree remarkably well with the *k*-seq results. Although our simulations cannot sample the entire conformational space of the *glmS* ribozyme, owing to its size (200 nt), the trajectories illustrate how single base substitutions in the core and IL4 directly or indirectly alter the interactions surrounding the active site.

First, we find that most deleterious mutations reduce activity by compromising self-assembly rather than by impairing catalysis or co-factor binding. This observation agrees with the finding of Andreasson et al (35) that harmful core mutations correlate with reduced *k*_cat_. We find that poor self-cleavage most often arises from a large inactive sub-population rather than slow cleavage of the whole population. The importance of self-assembly for ribozyme fitness is reinforced by our observation that detrimental mutations in the core map to base paired residues in P2–P2.1–P2.2 rather than unpaired residues (Figure 2A and Supplementary Figure S6). This is not due to weak secondary structure per se, as mismatch mutations in the peripheral P4 and P4.1 helices were not deleterious. Instead, the sensitivity of core helices to mutation can be rationalized by a competition between the core double-pseudoknot and less compact secondary structures that makes the core vulnerable to misfolding. This view is supported by force denaturation showing that the ribozyme core is marginally stable (31), and co-transcriptional folding in which the core pseudoknots are only kinetically favored within a short elongation window (55). Competition between core pseudoknots and less compact structures was also observed in group I ribozymes (64), RNase P (65), HDV ribozymes (66,67) and the 30S ribosome (68), suggesting that pseudoknots are an advantageous yet high-maintenance feature of functional RNA.

Second, mutations in the conserved P2.1 and P2.2 helices disrupt an intricate network of interactions required to position the GlcN6P co-factor and the scissile phosphodiester for self-cleavage (25,56,69). Our all-atom MD simulation results illustrate why substitution of just one core residue, A40, is so deleterious: G40 creates a new set of non-native hydrogen bonds that puts the cleavage site into an inactive conformation. We anticipate that a pyrimidine at position 40 and other mutations in P2.1 will similarly disrupt core tertiary interactions. Because the native hydrogen bonding network depends on many residues, mutations in any of them renders the ribozyme non-functional (Figure 2), even in high GlcN6P (35). Misfolding of the entire core likely occurs at longer times not accessible to our simulations.

Third, our results explain why the purine stack in internal loop IL4 is strictly conserved among *glmS* ribozymes (30). In the full-length *glmS* ribozyme, IL4 reinforces the P2.1 helix through a web of minor groove interactions (25). In agreement with this, we find that A114 and A139 at the center of the purine stack are most sensitive to substitution. Surprisingly, A114 and A139 mutations are inactive not because they weaken the core-IL4 interface, but because they create a new interface that corrupts the structure of the core. Biochemical evidence for this interpretation comes from improved activity of A114 and A139 variants at higher temperatures that weaken the core-IL4 interface, or when IL4 docking is blocked (Figure 5). Our MD simulations illustrate in detail how base substitutions in IL4 pull key residues such as G39 out of the core by enabling non-native hydrogen bonding interactions (Figure 7). Non-native contacts between G40 and IL4 were also observed in one A40G trajectory (Figure 6).

Lastly, our results support the idea that cooperative alignment of helical domains via peripheral tertiary interactions enhances ribozyme fitness (7,70). In the *glmS* ribozyme, the P4 domain is oriented with respect to the core by the P3 pseudoknot on one end and docking of the L4 tetraloop against its P1 receptor on the other end. Residues involved in these long-range tertiary interactions are intolerant to substitution (Figure 2 and (33,35)), whereas adjacent helices are not. The dual role of peripheral domains in the *glmS* ribozyme parallels the situation in group I ribozymes, where peripheral domains support self-assembly overall by aligning core helices (7) and specifically stabilize the active site (3,10,71,72). It will be interesting to learn how peripheral elements reshape the fitness landscape of ribozymes under physiological conditions of molecular crowding and low Mg^2+^ that favor generic self-assembly but not catalysis or ligand recognition (18,73). Our results highlight the degree to which the need to avoid non-native structure drives fitness in functional RNAs, which has also been observed in fitness landscapes of small artificial ribozymes (38).

## Supplementary Material

gkae830_Supplemental_Files

## Data Availability

Data associated with this study are available from the JHU Data Services Repository https://doi.org/10.7281/T1/JDHWJN and NCBI GEO Series accession number GSE261357.
