# Supplementary material for: Optimized periphery-core interface increases fitness of the Bacillus subtilis glmS ribozyme
Source: Nucleic Acids Res. 2024 Sep 25;52(21):13340–50. doi: 10.1093/nar/gkae830 (PMC11602151; doi:10.1093/nar/gkae830)
Supplement: gkae830_Supplemental_Files [file gkae830_supplemental_files.zip › Yu_glmS_paper1_SI-rev2.pdf]

SUPPLEMENTAL INFORMATION

Optimized periphery-core interface increases fitness of the *Bacillus subtilis glmS* ribozyme

Li-Eng Yu[1], Elise N. White[2], Sarah A. Woodson[3]*

[1]Cell, Molecular and Developmental Biology and Biophysics Program, Johns Hopkins University, Baltimore, MD 21218 USA

[2]Program in Molecular Biophysics, Johns Hopkins University, Baltimore, MD 21218 USA

[3]T.C. Jenkins Department of Biophysics, Johns Hopkins University, Baltimore, MD 21218 USA

*Correspondence to swoodson@jhu.edu

Supplemental Figures S1-S12

Supplemental Tables S1-S6

Supplemental Methods

Supplemental References

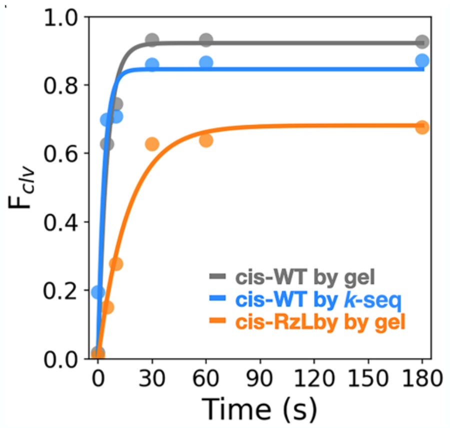

FIGURE S1. *k*-seq accurately measures the activity of the parental *cis-glmSCC* ribozyme. The self-cleavage kinetics of the parental "WT" Bsu *glmS* ribozyme measured by *k*-seq (light blue) agree well with activity measured by a conventional PAGE assay (gray; 1 of 4 trials shown). The input pool of ribozyme variants, RzLby, is less active than the parental RNA (orange; 1 of 3 trials), consistent with the presence of deleterious mutations. See Table S2 for observed rate constants and amplitudes. WT by

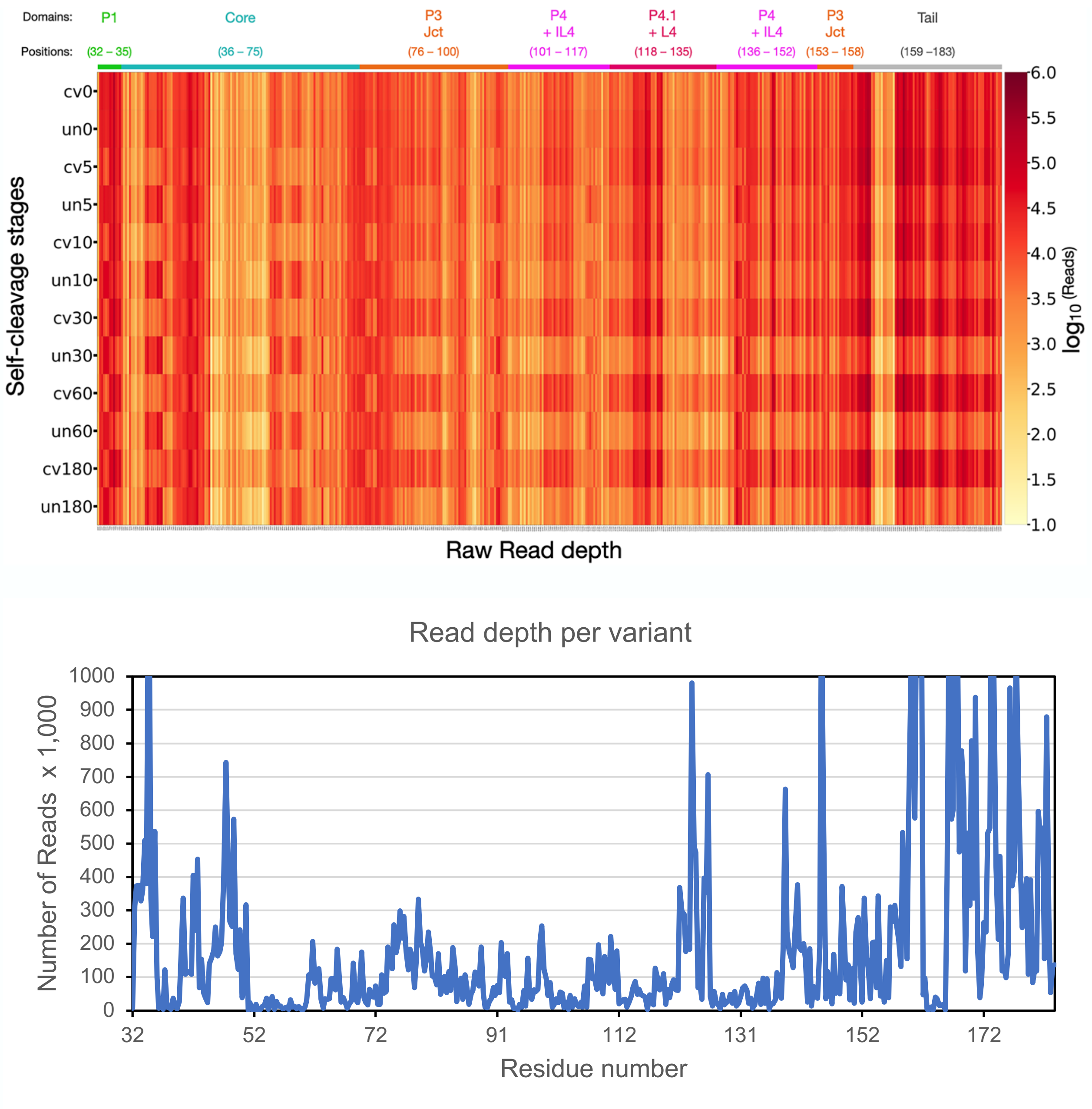

FIGURE S2. Read depth for all single mutations. Top, heatmap showing the log10 scale of 41,624,025 reads after quality filtering for individual single mutations in the 12 sub-libraries derived from each self-cleavage stage; each reaction time (0-180 s) is associated with a cleaved (cv) and uncleaved (un) RNA pool. The top color bar indicates the ribozyme domains, colored as in Figure 1. Median read depth = 6851 per variant per pool. Bottom, total reads (cv + un) per variant. Regions with low coverage per variant are due to secondary structure in the ssDNA template that prevents efficient annealing of oligonucleotides during mutagenesis. However, the minimum coverage (102 reads/variant at each reaction time) was still sufficient to determine the activity of individual variants, which was based on the fraction cleaved at six different time points.

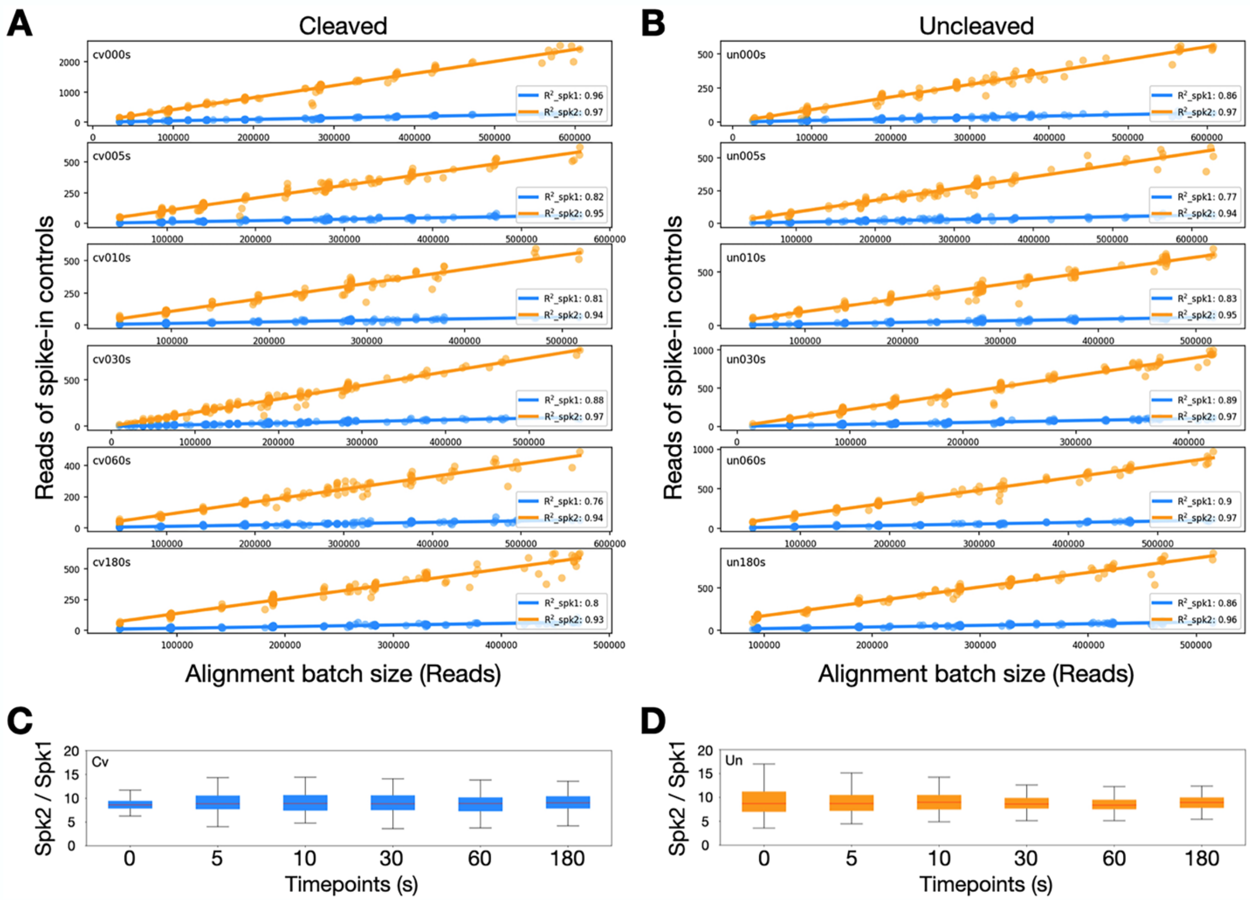

FIGURE S3. Estimating absolute RNA concentration using spike-in control RNAs. Two barcoded versions of the WT ribozyme were added to the purified RNA pools as spike-in controls, in a ratio 1:8 spk1:spk2 (see Methods). (A,B) The reads of spk1 and spk2 were counted in the individual alignment batch files that each contain a random number of selected reads. The count of spk1 and spk2 increased linearly as a function of alignment batch size (the total number of reads in each alignment file), with linear regressions among all sub-libraries yielding R^2 = 0.84 ± 0.06 for spk1 and 0.96 ± 0.02 for spk2 (mean ± SD). The linear regression coefficients were used to calculate the adjusted number of spk1 or spk2 reads for a given sub-library expected for a specified number of total aligned reads. (C, D) The distribution of the ratio of spk2 reads to spk1 reads in individual alignment batches of each sub-library for (C) cleaved RNA and (D) uncleaved RNA. The red solid line indicates the median of the ratio of spk2 to spk1, which remains constant across self-cleavage reaction times.

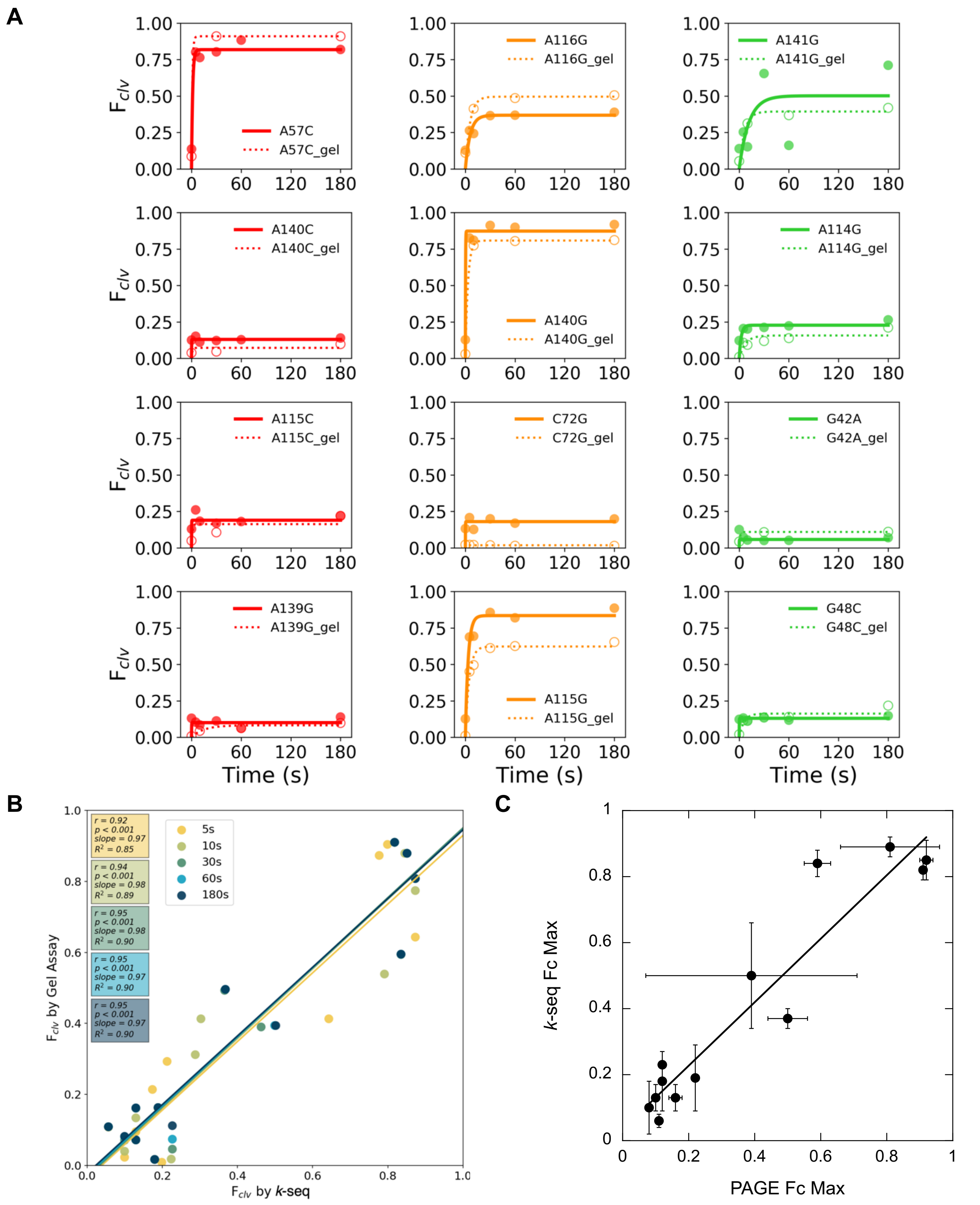

FIGURE S4. Sequencing accurately measures ribozyme variant activity. A. Comparison of observed self-cleavage kinetics of 12 selected variants measured by PAGE (dashed lines) and *k*-seq (solid lines) at 37 ˚C. Plots show one of 1-3 trials. See Table S2 for kinetics parameters. The observed rates of cleavage are difficult to estimate for residues with very rapid self-cleavage or a

very low amplitude of cleavage although the amplitudes correlate well for most residues. For certain residues such as C72G, *k*-seq overestimates the product yield relative to PAGE because of background cleavage at time = 0. Differences between the two assays are small for most variants, and do not apply to all base substitutions at a given position (for example, compare A115C and A115G). B. Correlation of fraction cleavage (F_{clv}) measured by PAGE and by *k*-seq. Parameters for linear regression of points at each reaction time are shown in the key; p-values from Pearson correlation (two-sided). **C.** Correlation of cleavage amplitudes measured by *k*-seq and PAGE. Error bars represent the statistical uncertainty in the fitted amplitudes. The slope of the linear regression is 0.96, with $R^2 = 0.95$. Correlation of rate constants (fewer comparisons) resulted in a slope of 1.25 with $R^2 = 0.55$.

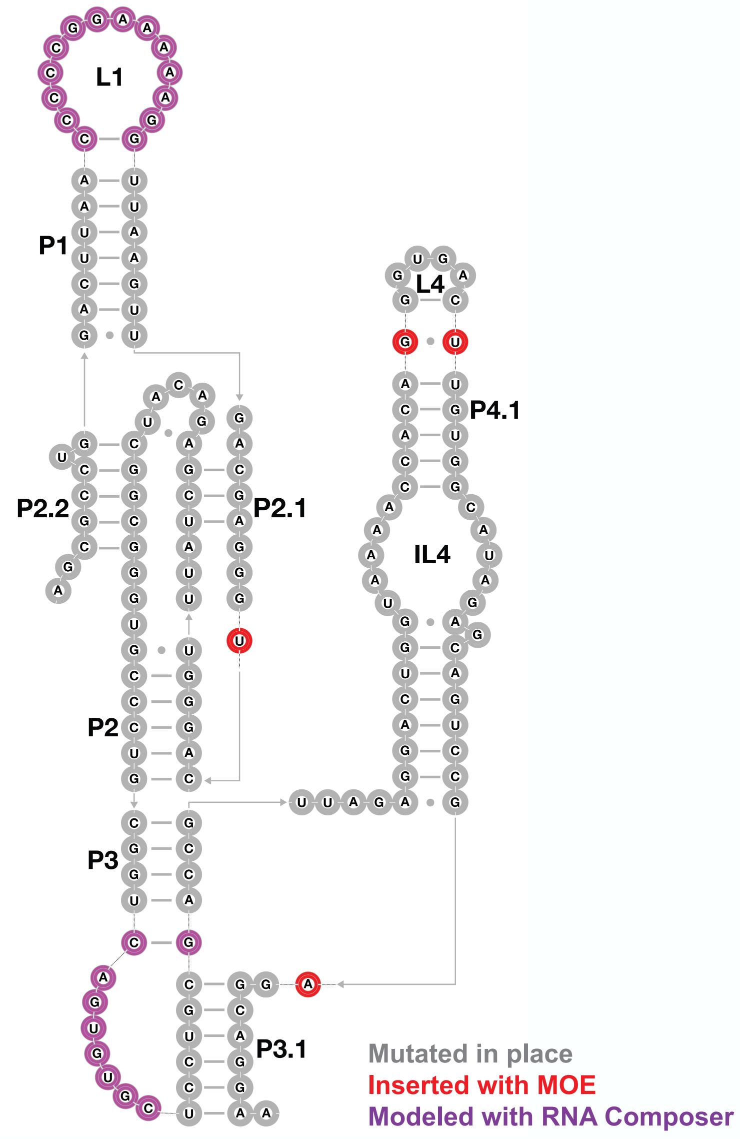

FIGURE S5. Building a *Bsu* ribozyme homology model. Initial coordinates of the 3D structure of the Bsu *glmS* ribozyme were constructed using the *Tte glmS* ribozyme crystal structure (PDB 2H0Z) as a template (1). Conserved regions (gray) were mutated in place as required to convert the *Tte* sequence to the *Bsu* sequence. Red bases were inserted using the RNA Builder tool in Molecular Operating Environment. Purple fragments were modeled as 2D fragments with RNA Composer (2) and docked with the main model by aligning overlapping base pairs. See Methods for details.

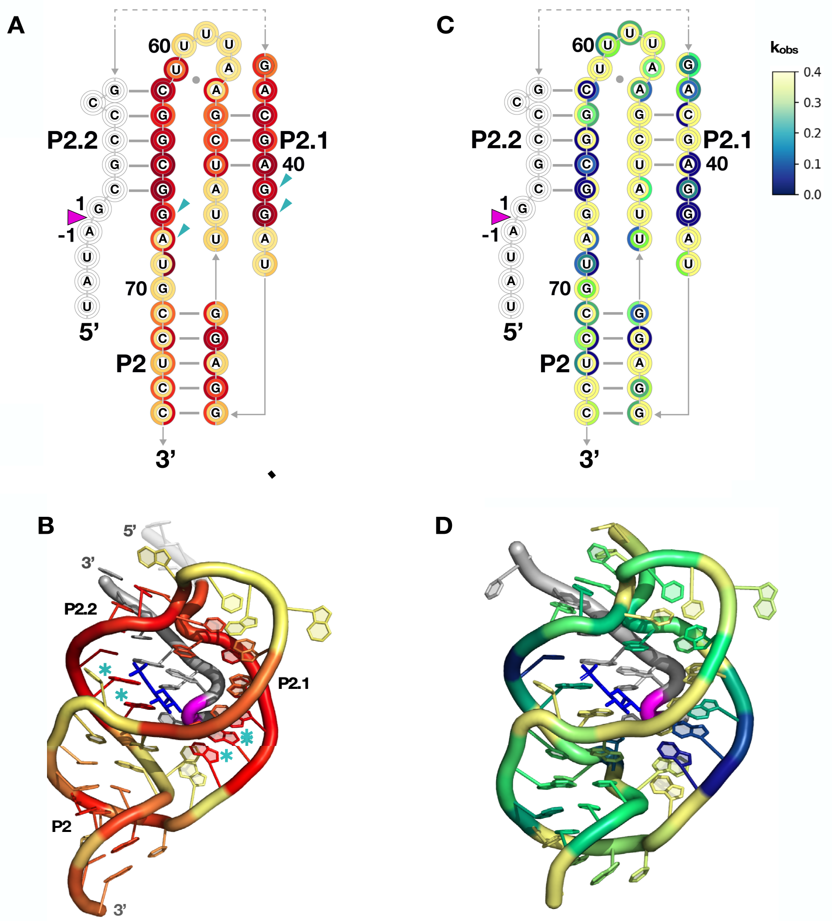

FIGURE S6. Mutations in the catalytic core reduce catalytic activity and ribozyme fitness. Related to Figure 2. (A) Expanded 2D heatmap of activity for the core region based on the yield of cleaved RNA at 180 s. Notation and color scheme as in Figure 2A, with red indicating low activity and yellow high activity. Parameters can be found in Appendix 1 (Dataset 1). (B) Expanded 3D heatmap showing how single base substitutions in the core P2-P2.2 helices reduce the yielded of active RNA (red) more than mutation in loops (yellow). Colors in the 3D map represent the average activity of the three base substitutions at each position. Green arrowheads in (A) and asterisks in (B) indicate residues that position the GlcN6P co-factor. (C,D) Heatmaps as in panels A,B showing that mutations in the co-factor binding pocket and core loops lower the rate constant for self-cleavage (s^{-1}) in the presence of saturating (10 mM) GlcN6P. Residues with an extent of cleavage ≤ 7% were assigned a rate constant of ≤ 0.01 s^{-1} (dark blue).

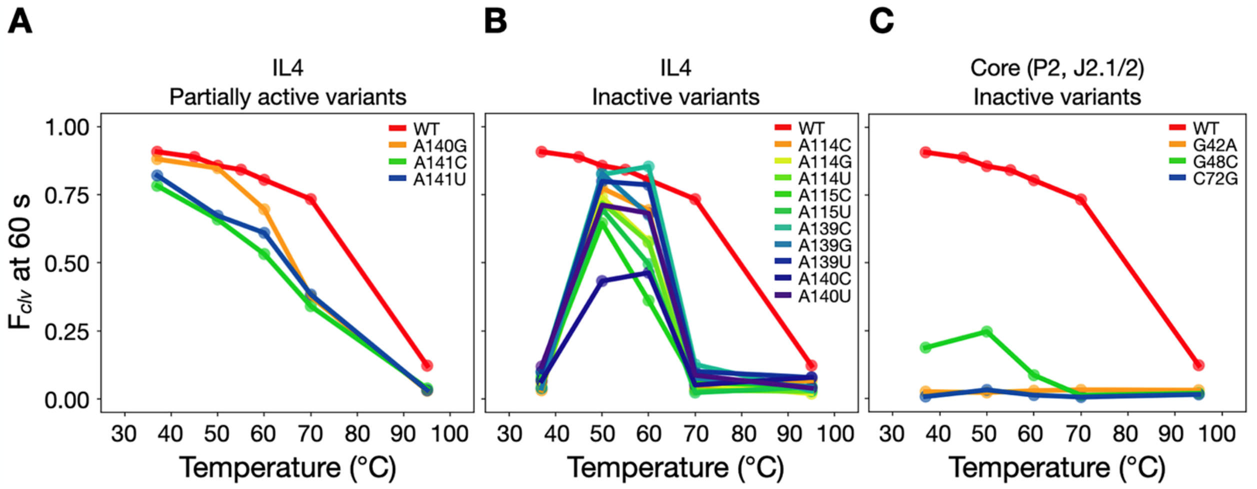

FIGURE S7. Misfolded IL4 structure is metastable. (A) Fraction cleaved at 60 s for partially active IL4 variants measured at varying temperatures (1 trial), compared to the parental ribozyme (red). (B) The same as in panel A but for IL4 variants showing little or no self-cleavage at 37 °C and normal activity at 50 ˚C. (C) The same as in panel A but for deleterious core variants with mutations in the P2 helix or J2.1/2 segment. Related to Figure 4.

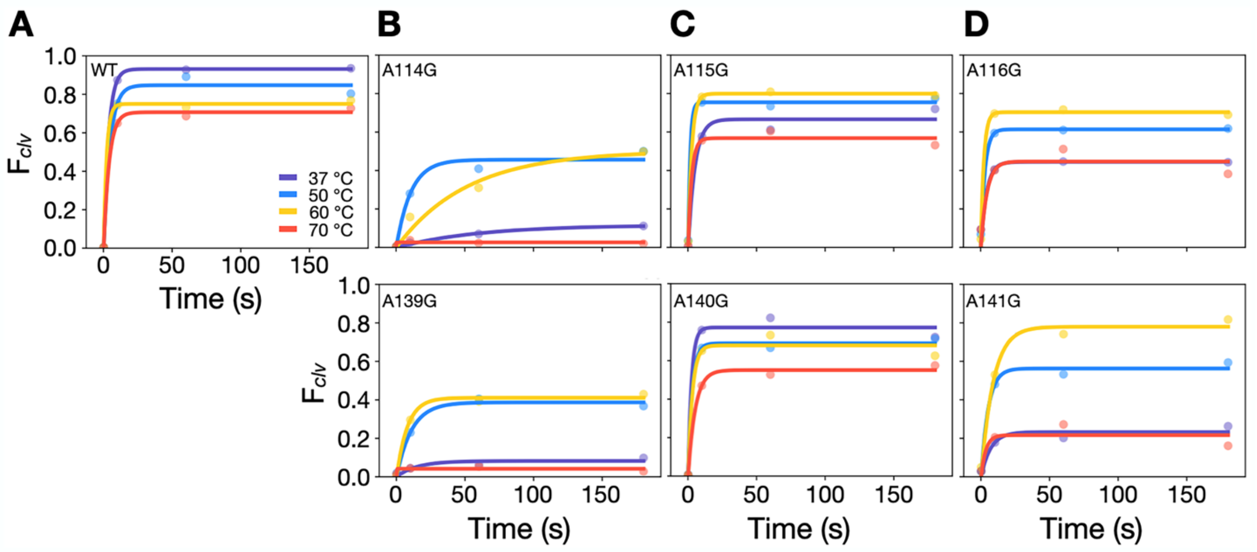

FIGURE S8. Heat increases the yield but not the self-cleavage rate of IL4 ribozyme variants. The kinetics of self-cleavage for WT (A) and 6 selected IL4 mutants (B – D) were measured at different temperatures by PAGE (1 trial). The data were fitted by a single exponential equation. Parameters can be found in Table S3. Most mutations affected the endpoint of the reaction, consistent with a change in the amount of native ribozyme.

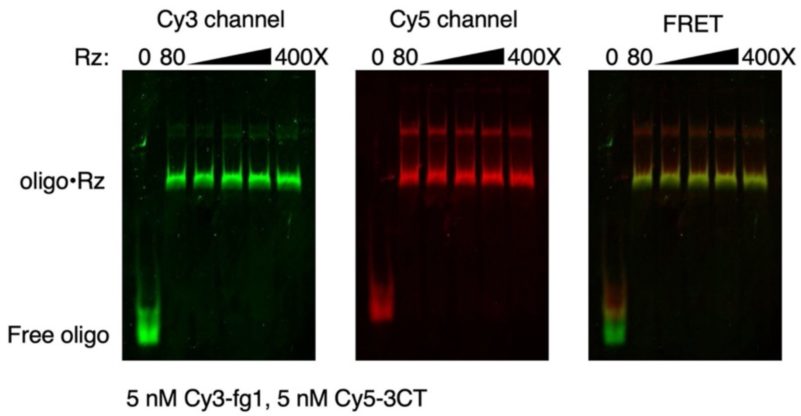

FIGURE S9. Binding of 3CT control oligonucleotide to the 3′ end of the Bsu *glmS* ribozyme. Trans-cleaving version of the ribozyme RNA (Rz) was annealed with 5 nM each Cy3-fg1 substrate RNA and 5 nM Cy5-3CT oligonucleotide complementary to the 3′ tail that was used as a control in Figure 5. RNA was added in 80 to 400-fold excess. Samples were separated by native PAGE and the gels were imaged with a Typhoon 9410 scanner. Three exposures of the same native gel, showing Cy3 intensity, Cy5 intensity and fluorescence resonance energy transfer.

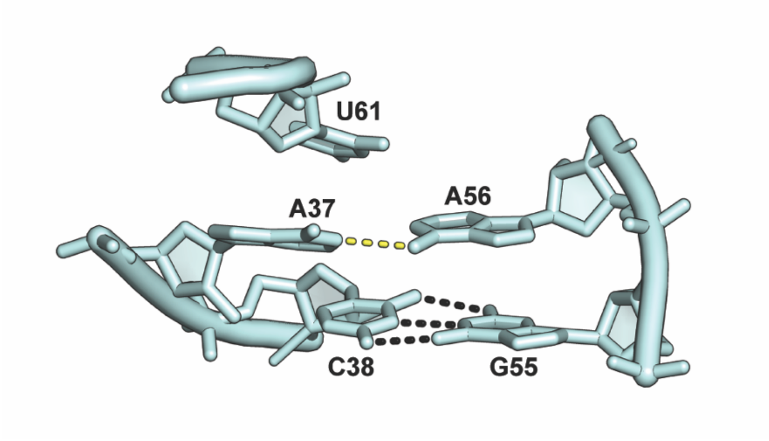

FIGURE S10. The conserved A56-U61 base pair, which stacks on top of the A37*(C38-G55) recognition triple, was replaced by a non-native hydrogen bond (yellow) between A37 and A56 in 64% of frames in a perturbed A40G replica.

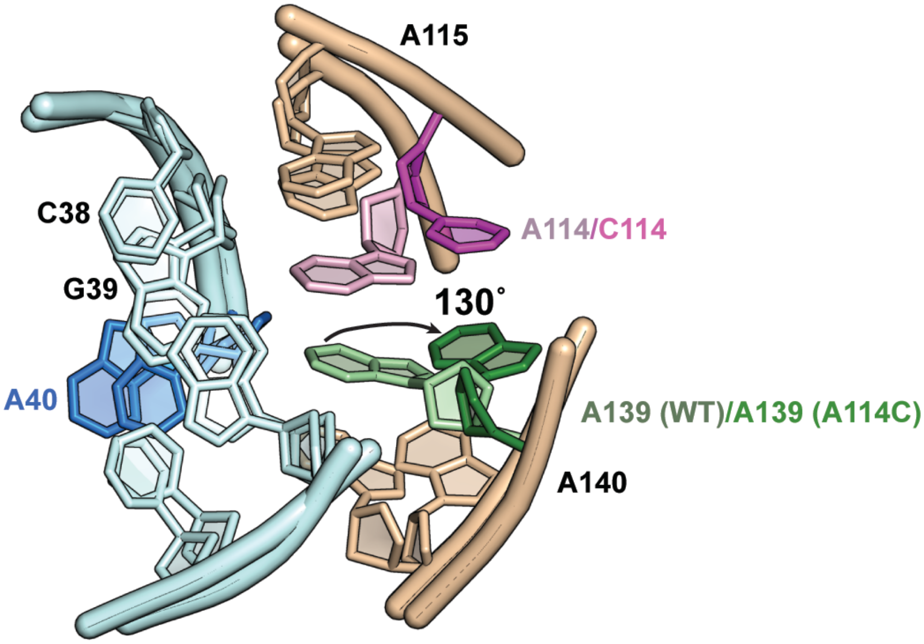

FIGURE S11. In 7/10 A114C replicas, IL4 undocks from the core. This can partly be explained by A139 swiveling by 130˚ to stack underneath C114, which is positioned further from the minor groove of the P2.1 helix than A114.

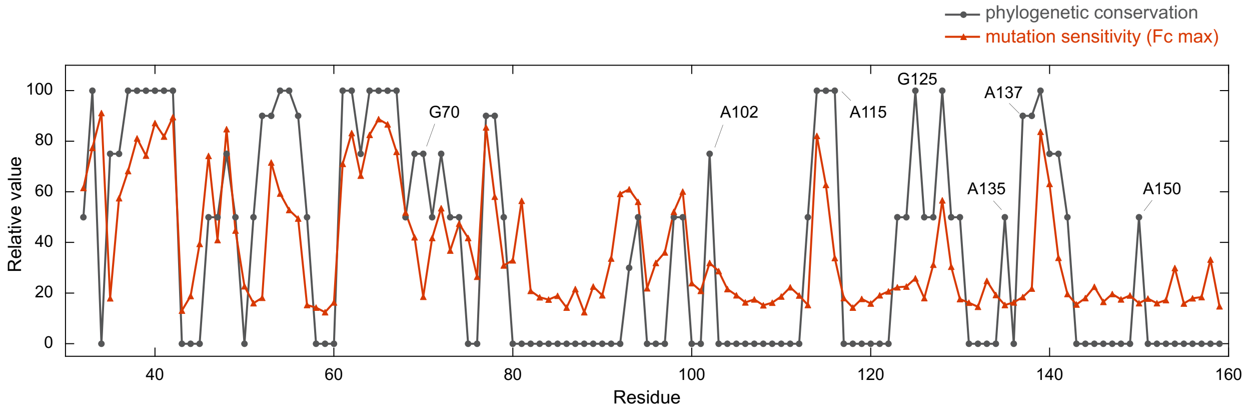

FIGURE S12. Comparison of phylogenetic sequence conservation (grey) and the activity map of the *Bsu glmS* ribozyme (red). For this qualitative comparison, the conservation of nucleotide identity at each position according to McCown et al (3) was ranked as follows: 97% identical, score 100, 90% identical, score 90, 75% identical, score 75, R or Y >75%, score 50. All other residues were assigned score 0. The presence (or absence) of a nucleotide was not considered because our variant pool did not include insertions and deletions. The sensitivity of each position in the *Bsu glmS* ribozyme to base substitution in the *k*-seq data was determined from $1 - \langle F_C \rangle$, in which $\langle F_C \rangle$ is the average cleavage amplitude of the three base substitutions. For indicated positions with poor agreement, at least one of the three possible base substitutions results in slow cleavage, suggesting the overall agreement between self-cleavage activity and conservation is very good overall.

Table S1: Sequences of DNA primers and oligonucleotides used in this study.

Name	Sequence	Source
cis-gSCCop19clv-nPCRF1a	GAACTGGGCCCCCGGAAAAAGGCCC	IDT
cis-gSCCop19clv-nPCRF2a	TCAGCCTAATACGACTCACTATAGGCTTTACCTATAATTATAGCGCCCGAACTGGGCCCC	
PF-Rv	GCGGATAACAATTTCACACAGGAAACAGC	
gS-1stPCR-F	TCGTCGGCAGCGTCAGATGTGTATAAGAGACAGGCGCCCGAACTGGGCCCCCGGAAAAAG	
gS-1stPCR-R	GTCTCGTGGGCTCGGAGATGTGTATAAGAGACAGGCGGATAACAATTTCACACAGGAAAC	
Mutagenesis oligonucleotide library	30 bases complementary to the ribozyme sequence, with a degenerate residue N at the center.	

Table S2: Cleavage kinetics of the *Bsu glmS* ribozyme by *k*-seq and PAGE.†

	***k*-seq**		**PAGE**	
**RNA**	**k_{obs} (s^{-1})**	***A***	**k_{obs} (s^{-1})**	***A***
WT_*k*-seq	0.30 ± 0.12	0.85 ± 0.06	0.20 ± 0.02	0.92 ± 0.02
RzLby			0.06 ± 0.01	0.68 ± 0.03
G33U	#	0.10 ± 0.04	n.d.§	
G42A	#	0.06 ± 0.03	‡	0.11 ± 0.03
G48C	#	0.13 ± 0.03	0.18 ± 0.10	0.16 ± 0.02
A57C	0.75 ± 0.94	0.82 ± 0.04	‡	0.91 ± 0.06
A68U	0.02 ± 0.01	0.44 ± 0.12	n.d.	
C72G	#	0.18 ± 0.03	#	0.02 ± 0.01
U92C	0.13 ± 0.04	0.74 ± 0.06	n.d.	
A114G	0.42 ± 0.51	0.23 ± 0.03	0.14 ± 0.03	0.16 ± 0.02
A115C	#	0.19 ± 0.03	‡	0.16 ± 0.07
A115G	0.30 ± 0.10	0.84 ± 0.05	0.24 ± 0.08	0.62 ± 0.02
A116G	0.17 ± 0.09	0.37 ± 0.04	0.18 ± 0.11	0.50 ± 0.06
A128G	0.50 ± 0.42	0.45 ± 0.04	n.d.	
A139G	#	0.1 ± 0.04	0.07 ± 0.05	0.08 ± 0.01
A140C	#	0.13 ± 0.03	‡	0.07 ± 0.04
A140G	0.49 ± 0.14	0.87 ± 0.04	0.32 ± 0.08	0.81 ± 0.02
A141G	0.09 ± 0.10	0.50 ± 0.16	0.16 ± 0.06	0.39 ± 0.03

† Observed rate constants at 37 ˚C were estimated from reads in cleaved and uncleaved RNA pools (*k*-seq) or from the relative intensity of cleaved and uncleaved RNA in denaturing gels (PAGE). *A* is the amplitude of the cleavage reaction. The errors represent one standard deviation of the parameters obtained by least squares fits of an exponential rate equation to the data for one trial (see Methods) or three trials for A114G and A115C by PAGE.

# k_{obs} could not be reliably determined due to low activity or high background at t = 0. These variants were either inactive (G33U, G42A), or reactive to a low extent (A139C, A140C).

‡*F*c was only measured at 0, 30 and 180 s.

§n.d., Not done.

Table S3: Comparison of the cleavage kinetics at different temperatures by PAGE.

	37 ˚C			50 ˚C			60 ˚C			70 ˚C		
**RNA**	k_{obs} (s^{-1})	*A*	**Yield 180 s**	k_{obs} (s^{-1})	*A*	**Yield 180 s**	k_{obs} (s^{-1})	*A*	**Yield 180 s**	k_{obs} (s^{-1})	*A*	**Yield 180 s**
WT	0.28 ± 0.01	0.93 ± 0.01	0.93 ± 0.01	0.22 ± 0.06	0.85 ± 0.03	0.85 ± 0.03	0.47 ± 0.31	0.75 ± 0.01	0.75 ± 0.01	0.25 ± 0.04	0.71 ± 0.01	0.71 ± 0.01
A114G	0.02 ± 0.01	0.12 ± 0.01	0.11 ± 0.01	0.09 ± 0.03	0.46 ± 0.03	0.46 ± 0.03	0.06 ± 0.03	0.56 ± 0.08	0.61 ± 0.07	2.16 ± 0.01	0.03 ± 0.01	0.03 ± 0.01
A115G	0.20 ± 0.07	0.67 ± 0.04	0.67 ± 0.04	0.70 ± 5.82	0.76 ± 0.02	0.76 ± 0.02	0.39 ± 0.18	0.80 ± 0.02	0.80 ± 0.02	0.39 ± 0.39	0.57 ± 0.03	0.57 ± 0.03
A116G	0.24 ± 0.20	0.45 ± 0.05	0.45 ± 0.05	0.34 ± 0.30	0.61 ± 0.03	0.61 ± 0.03	0.46 ± 0.56	0.70 ± 0.02	0.70 ± 0.02	0.23 ± 0.24	0.45 ± 0.07	0.45 ± 0.07
A139G	0.07 ± 0.05	0.08 ± 0.01	0.08 ± 0.01	0.09 ± 0.02	0.39 ± 0.02	0.39 ± 0.02	0.13 ± 0.02	0.41 ± 0.01	0.41 ± 0.01	3.46 ± 0.01	0.04 ± 0.01	0.04 ± 0.01
A140G	0.41 ± 0.45	0.77 ± 0.04	0.77 ± 0.04	0.33 ± 0.12	0.69 ± 0.02	0.69 ± 0.02	0.32 ± 0.24	0.68 ± 0.04	0.68 ± 0.04	0.19 ± 0.03	0.55 ± 0.02	0.55 ± 0.02
A141G	0.15 ± 0.08	0.23 ± 0.03	0.23 ± 0.03	0.19 ± 0.05	0.56 ± 0.03	0.56 ± 0.03	0.11 ± 0.02	0.78 ± 0.04	0.78 ± 0.04	0.29 ± 0.61	0.22 ± 0.04	0.22 ± 0.04

Table S4: Cleavage kinetics at 37 ˚C in the presence of anti-IL4 oligonucleotides.‡

	–Anti-IL4		+Anti-IL4	
**RNA**	k_{obs} (s^{-1})	*A*	k_{obs} (s^{-1})	*A*
WT	0.42 ± 0.15	0.91 ± 0.02	0.02 ± 0.01	0.64 ± 0.03
A140G	1.08 ± 0.65	0.78 ± 0.02	0.02 ± 0.01	0.57 ± 0.01
A114G	0.06 ± 0.03	0.12 ± 0.01	0.02 ± 0.01	0.50 ± 0.05
A139G	0.05 ± 0.01	0.10 ± 0.01	0.02 ± 0.01	0.57 ± 0.06
G48C	0.89 ± 0.74	0.19 ± 0.03	0.68 ± 0.62	0.03 ± 0.01
C72G	3.45 ± 1.41	0.03 ± 0.01	1.37 ± 0.71	0.02 ± 0.02
WT + anti-3CT	1.00 ± 0.76	0.99 ± 0.01		

‡ Measured by PAGE as described in Methods. Values are the mean and ± SEM of three independent experiments.

Table S5. Summary of molecular dynamics simulations performed.

Run No.	Model	Purpose	Length of Simulation (ns)
1	Homology Model	Full Restraint Equilibration (Optimization)	20
2	Optimized Model (WT) Rep 1 – Rep 10	Minimal Restraint Production	50 (x10)
3	Optimized Model (A40G) Rep 1 – Rep 10	Minimal Restraint Production	50 (x10)
4	Optimized Model (A114C) Rep 1 – Rep 10	Minimal Restraint Production	50 (x10)
Total			1,520

Table S6. Hydrogen bonding interactions among selected core and IL4 residues.[a]

	Core + Cleavage Site		Core + IL4		
**Nts:**	G1, A-1, C54, G41, G67			38-40, 55, and 114, 139, 140	
	**Replica**	**% Total Frames Present**[b]		**Replica**	**% Total Frames Present**
**WT**			**WT**		
	1	94		1	99
	2	99		2	95
	3	84		3	99
	4	83		4	97
	5	95		5	99
	6	99		6	36
	7	99		7	99
	8	99		8	100
	9	97		9	99
	10	81		10	100
**A40G**			**A114C**		
	1	98		1	68
	2	90		2	80
	3	13		3	77
	4	99		4	70
	5	98		5	99
	6	3		6	70
	7	99		7	61
	8	12		8	99
	9	99		9	97
	10	99		10	99

[a]Number of hydrogen bond interactions relative to possible hydrogen bonds between selected residues for each region analyzed.
[b]Fractional occupancy of hydrogen bonds for each group of residues was calculated as the number of hydrogen bonds per frame summed overall all frames, relative to the possible hydrogen bonds summed over all frames in the trajectory.

Table S7. Weak restraints applied during MD simulations.

Distance Restraints Applied During Equilibration

Base Pairs		*Tertiary Contacts*	
Atom 1	Atom 2	Atom 1	Atom 2
66G H1	2C N3	12G H4’	126U N3
7G H1	62C N3	1G H2’1	39G O6
8A N1	35U H3	1G H2’1	39G O6
28G H1	15C N3	54C H42	39G O6
55G H1	38C N3	37A N3	38C H41
39G H1	54C N3	57A H62	138C O2’
40A N1	53U H3	142G O6	53U H4’
45G H1	75C N3	142G N7	53U H4’
49G H1	71C N3	137A H62	116A N7
56A N1	61U N3	137A N1	116A H61
100G H1	76C N3	138C O2	115A H61
96A N1	80U H3	115A N6	114A H2’1
81G H1	95C N3	115A O4’	114A H2’1
89A N1	158U H3	114A H62	56A O4’
94A N1	153U H3	114A N1	55G H22
104G H1	151C N3	139A N1	39G H22
105G H1	149C N3	139A H62	40A O4’
146A N1	108U H3		
143A N1	111U H3		
141A N1	112U H3		
135G H1	118C N3		
121G H1	132C N3		
124G H1	129C N3		
119A N1	134U H3		

Angle Restraints Applied During Equilibration

Atom 1	Atom 2	Atom 3
57A N3	57A O4’	57A C2’
57A N9	57A O4’	57A C2’

Distance Restraints Applied During Production

Base Pairs		*Tertiary Contacts*	
Atom 1	Atom 2	Atom 1	Atom 2
66G H1	2C N3	12G H4’	126U N3
8A N1	35U H3	1G H2’1	39G O6
28G H1	15C N3	1G H2’1	39G O6
39G H1	54C N3	54C H42	39G O6
45G H1	75C N3	37A N3	38C H41
49G H1	71C N3	142G O6	53U H4’
100G H1	76C N3	142G N7	53U H4’

Table S7 cont.

96A N1	80U H3
81G H1	95C N3
89A N1	158U H3
94A N1	153U H3
104G H1	151C N3
105G H1	149C N3
146A N1	108U H3
143A N1	111U H3
141A N1	112U H3
135G H1	118C N3
121G H1	132C N3
124G H1	129C N3

SUPPLEMENTAL METHODS

Preparation of ribozyme variant library

The parental *glmS* ribozyme sequence (4) was amplified from *B. subtilis* strain UD1022 locus CP011534 (nucleotide 501 to 670). The amplicon, including a T7 promoter and terminator and the mutation U31C, was cloned into the *BssHII* restriction site of pBlueScript II SK(-) to yield pBlueScript II SK(-)-*BbvcI-trans-glmSCC*. The first nucleotide transcribed by T7 RNA polymerase is G13.

Plasmid pBlueScript II SK(-)-*BbvcI-trans-glmSCC* was used to generate the initial library of ribozyme variants by nicking mutagenesis (5). 152 consecutive nucleotides, from A32 to C183, were targeted for mutagenesis by 152 complementary oligonucleotides 27-40 nt long carrying one random nucleotide (25% each base) at the center (IDT with standard desalting purification). The mutagenic oligonucleotides were separated into two groups based on the predicted secondary structure (stem or loop) of the anti-sense single-stranded DNA template. To reduce competition between overlapping oligonucleotides targeting consecutive sites in the template, each group of oligonucleotides was divided into three subgroups with target sites three nucleotides apart. Mutagenic strands were synthesized separately for each of the six oligonucleotide subgroups according to the nicking mutagenesis protocol. The six product mixtures were purified and

combined into one reaction to generate the complementary strands. The final pool of variant plasmids was transformed into DH5α cells (NEB, C2987H) and grown on 10 LB agar plates supplemented with 100 μg/mL ampicillin selection plates (100 mm x 15 mm). All cells were scraped from the plates and pooled before plasmid isolation (QIAprep Spin Miniprep kit, 27104) to obtain the pBlueScript II SK(-)-*BbvcI-trans-glmSCC*-variant plasmid library.

***k*-seq library preparation**

DNA templates. A pool of cis-ribozyme variant DNA templates was amplified by two rounds of nested PCR (50 μL) from 1 ng pBlueScript II SK(-)-BbvcI-*trans-glmSCC*-variant plasmid library. The upstream sequence of *trans-glmSCC* was extended by 54 bp to restore the cleavage site (*glmS* 5′ UTR nt -18 to +12) and to add a T7 promoter and six additional base pairs). The first round of PCR was accomplished with forward primer *cis-gSCCop19clv*-nPCRF1a and reverse primer PF-Rv, while the second round of PCR used forward primer *cis-gSCCop19clv*-nPCRF2a and PF-Rv reverse primer. After the first round of 36 cycles, excess primers were digested with *ExoI* (NEB M0293S). The treated mixture was diluted 1000-fold and used as a template for the second round of PCR (36 cycles). The final product was purified (Monarch® PCR & DNA Cleanup). This *cis*-ribozyme DNA template encodes a 276-nt RNA from G(-18) to C183 of the *glmSCC* ribozyme sequence plus an additional G at the 5' end and a 74-nt 3′ extension.

Ribozyme RNA selection. The pooled cis-ribozyme variant DNA templates (10 μg) were transcribed *in vitro* without Tris buffer (6), and full-length transcripts (276 nt) were purified from a denaturing 6% polyacrylamide gel. Cleaved products were not detectable at this stage. For each reaction timepoint, 50 pmol purified ribozyme library was diluted into 15 μL reaction buffer (50 mM K-HEPES pH 7, 10 mM $MgCl_2$, 50 mM NaCl, 5 mM KCl). The RNA was denatured at 95 °C and snap-cooled on ice, then renatured by incubating 10 min at 70 °C followed by 15 min step-cooling to 37 ˚C (7). Self-cleavage was started by adding 3 μL 60 mM GlcN6P (10 mM final, pH 6.5-6.8). The cleavage reaction was quenched at 0, 5, 10, 30, 60, or 180 s by adding an equal volume (18 μL) 100 mM EDTA. For each timepoint, the cleaved and un-cleaved ribozyme were separated by 8 M urea 5% PAGE to generate a total of 12 RNA pools.

Sequencing. To estimate the absolute copy number of each variant and track bias in preparation of the cDNA library, two barcoded cis-*glmSCC* ribozymes (spk1, spk2) were

nominally added at $1 \cdot 10^{-4}$ ng/μL and $5 \cdot 10^{-4}$ ng/μL, respectively, as spike-in controls. 1 μL each spike-in control was blended with 1 μL 1 ng/μL from one post-cleavage ribozyme pool, yielding 3 μL RNA mixture for each of the 12 samples. Each RNA mixture was reverse-transcribed into a post-cleavage cDNA library with forward primer gS-1stPCR-F and reverse primer gS-1stPCR-R using SuperScript™ IV One-Step RT-PCR System kit (Invitrogen, 12594025). The cDNA library was amplified with dual Illumina compatible adapters with specific barcodes by following the nested PCR method B described in (8). The 12 individual barcoded post-cleavage cDNA sub-libraries were combined into an output library (152Lby) for sequencing via Illumina 150-bp paired-end (PE150) HiSeq (Novogene).

_k_-seq data analysis

Alignment. Deep sequencing of the 152Lby output library generated a total of 393,988,302 effective raw paired-end reads, with an average of 32,832,859 raw paired-end reads per each of the 12 samples (sub-libraries). Of this total, 12.6% (49,715,805 reads) were WT and 10.6% (41,624,025 reads) contained a single mutation. The average read depth for individual single mutants was 15,219. After filtering out low-quality sequencing reads (Q-score ≥ 21.82), mutations were identified by global alignment with the *cis-glmSCC* WT ribozyme sequence (Needleman-Wunsch).

The alignment was performed on batch files containing a random number of reads drawn sequentially from the HiSeq output. In most alignment batches, at least 90% reads contained no more than three mutations (Hamming distance < 4). Reads containing mutations were evenly dispersed within the HiSeq dataset. Additionally, WT reads were more prevalent in the cleaved RNA sub-libraries than in the uncleaved RNA sub-libraries, whereas reads containing ≥ 3 mutations displayed the opposite distribution.

Spike-in controls. Reads corresponding to the spike-in control RNAs (spk1 and spk2) were used to estimate the enrichment of individual sequences in the cleaved and uncleaved sub-libraries and track biases during the library preparation. The spike-in controls contained the *cis-glmS* WT ribozyme sequence, except that an 8-nt contig in the non-mutagenized 3′ region was replaced by a

unique barcode. As anticipated, the spk1 and spk2 ribozymes and parental WT ribozyme exhibited the same cleavage kinetics.

RNA concentration. For each RNA sample, a linear regression of the number of spk1 or spk2 reads in each alignment batch versus its corresponding batch size was used to estimate the number of spike-in reads for any sized pool of sequencing reads (Figure S2). The median ratio of spk2 to spk1 was similar in all 12 pools, corresponding to an actual ratio of 1:8 spk2:spk1. Assuming the number of reads per RNA concentration is the same for the variants and the spike-in RNAs for a given alignment file, the linear regression coefficients *m*, *b* for the spike-in reads were used to estimate the absolute concentration of each RNA variant in each sample pool (sub-library):

$$\frac{[variant_i]}{[spk2]} = \frac{(read\ count)_i}{m\ (total\ reads)+b} \qquad \text{(eq 1)}$$

in which $[variant]_i$ is the inferred concentration of a given sequence variant, $(read\ count)_i$ is the number of reads corresponding to variant *i* in the sub-library of aligned sequences, and (*total reads*) is the total number of reads in the sub-library. The fraction of each sequence variant *i* that was cleaved at a given timepoint was calculated as:

$$F_{c,i}(t) = \frac{[variant]_{cv,i}}{[variant]_{cv,i}+[variant]_{un,i}} \qquad \text{(eq 2)}$$

The notations $[variant]_{cv,I}$ and $[variant]_{un,i}$ represent the absolute concentration of $variant_i$ in cleaved and uncleaved samples for a given reaction time *t*. The results were fitted by a single exponential equation $F_c(t) = A \cdot \left(1 - e^{(-kt)}\right)$ to obtain the amplitude *A* and observed rate constant *k* of the self-cleavage reaction (scipy.optimize.curve_fit, (9)). The standard deviation of the values was taken from the square root of the diagonal of the variance-covariance matrix of the parameters.

Quality inspection. Random mutations which inevitably arise during library preparation and sequencing will lead to false mutation calling and contribute to error in the estimated of fraction of self-cleavage for individual ribozyme variants. Spike-in sequencing reads that ideally correspond to the WT ribozyme were used to estimate the sequencing error rate and remove low quality batches of reads. The median mutation rate for spk1 and spk2 reads was 0.1 % over the first 80% of 152Lby reads, but a few hot spots with mutation rates up to 2% were common to all 12 sub-libraries. The spk1 and spk2 mutants had little effect on self-cleavage and did not partition

into the cleaved and uncleaved pools as expected based on the phenotype of that variant. Therefore, mutations in the spike-in sequences were likely not present in the RNA, but most likely introduced during reverse transcription and library preparation.

To identify and exclude low quality batches of aligned sequencing reads with a high number of mutated spike-in controls, the fraction of spk1 and spk2 reads that had the correct (WT) sequence in the body of the ribozyme was calculated for each individual alignment batch, which is equivalent to the chance of retrieving a wild type spk1 or spk2 read. In each post-cleavage library, the mean fractions of WT spk1 and spk2 were similar but with a larger variation for spk1 (Figure S3). For each alignment batch, the fraction of reads with Hamming distance < 4 strongly correlated with the fraction of WT spk1 and spk2 reads, suggesting a greater probability of false mutation calling in alignment batch with higher mutation rates. Because nicking mutagenesis should rarely introduce more than three mutations into the same DNA template, reads with Hamming distance ≥ 4 may contain mutations introduced during library preparation or sequencing, and are likely invalid. To sort out the invalid reads, a quality filter algorithm was used to select good quality alignment batches by setting a cutoff corresponding to the fraction of valid reads (WT + variants with Hamming < 4) relative to the total number of reads in each alignment batch. A cutoff of 0.91 eliminated many poor-quality batches of reads while preserving at least 70% of the data and maintaining reasonable coverage for all the 12 sub-libraries.

Systematic error. The effects of non-random sequencing errors on mutation calling was modeled by assuming that the error frequencies of spk1 and spk2 represent that of the RNA pool, and that mutations at sites i and j are uncorrelated. Single mutations that convert the WT RNA to mutation i (false positive, FP) will contribute to an overestimate of F_{clv} for that mutant. Because we considered only single substitutions, a second mutation at site j in variant i will cause that read to be excluded from the counts for variant i (false negative, FN). For each variant i, $R_{cv}^{true} = R_{cv}^{exp} - R_{cv}^{FP} + R_{cv}^{FN}$ and $R_{un}^{true} = R_{un}^{exp} - R_{un}^{FP} + R_{un}^{FN}$. The false positives were estimated from the spike-in mutation frequency at each position, μ_i, weighted by the WT cleavage frequency χ_{cv}^{WT} and χ_{un}^{WT}, yielding $R_{cv,i}^{FP} = R_{cv,i}^{exp}(\chi_{cv}^{WT}\mu_i)$ and $R_{un,i}^{FP} = R_{un,i}^{exp}(\chi_{un}^{WT}\mu_i)$. The likelihood that a second mutation at site $j \neq i$ will falsely remove reads for variant i was estimated from the cumulative likelihood of two mutations in the ribozyme, $R_{cv,i}^{FN} = R_{cv,i}^{exp}(P(n,\lambda)$ and $R_{un,i}^{FN} = R_{un,i}^{exp}(P(n,\lambda)$, in

which $P(n,\lambda)$ is the upper incomplete gamma distribution, $n = 2$ and $\lambda = N\langle\mu_i\rangle$ equals the average mutation rate over a 152 nt sequence. Combining these expressions leads to

$$F_{clv,i}^{true} = \frac{F_{clv,i}^{exp}}{F_{clv,i}^{exp} + \alpha\left(1 - F_{clv,i}^{exp}\right)} \qquad \text{and} \qquad \alpha = \frac{\left(1 - \chi_{un}^{WT}\mu_i + P(n,\lambda)\right)}{\left(1 - \chi_{cv}^{WT}\mu_i + P(n,\lambda)\right)} \qquad \text{(eq 3)}$$

Assuming a 2.5% error rate, which was the maximum we observed at any position, and an average of 0.1% per site, the error in $F_{\mathrm{clv,i}}$ expected from this source of false mutation calling is 1-2%. This source of error would have a negligible effect on the fitted cleavage rates.

Self-cleavage activity by PAGE

For the cis-ribozyme, 5 pmol RNA was heat-denatured at 95 °C for 5 min in 2.5 μL HK buffer followed by snap-cooling on ice for 5 min. The ribozyme was then diluted 20-fold in 50 μL HMN buffer containing 10 mM Mg^{2+} for 70 °C renaturation followed by the step-cooling protocol above. The cleavage reaction was initiated by 10 mM GlcN6P (final) and halted by 50 mM EDTA at each desired timepoint. The cleaved and un-cleaved ribozyme (19 nucleotide difference between these two RNA species) was separated by 5% Urea-PAGE. The gel was stained with SYBR-Gold (1:100,000 in 1X TBE) and scanned (Amersham Typhoon 5 Biomolecular Imager). The fraction of cleaved cis-ribozyme was quantified by ImageJ and fitted to a single exponential equation. For the ribozyme cleavage assay with anti-IL4 RNA oligomers, 0.5 pmol *cis*-ribozyme was premixed with 200 pmol each of the two RNA oligonucleotides before renaturation (400:400:1 molar ratio). The remaining procedures for estimating the cleavage kinetics were the same as described above.

For ribozyme cleavage assays at temperatures ranging from 37 to 95 °C, 2 pmol *cis*-ribozyme was pre-folded in buffer containing 10 mM Mg^{2+} as described above, then incubated at the desired temperature for at least 3 min. Next, 10 mM GlcN6P (final concentration) was mixed with the RNA at the reaction temperature to initiate self-cleavage. The reaction was stopped with 50 mM EDTA (final concentration) at several timepoints to measure the cleavage kinetics as needed. The remaining steps for quantifying fraction cleave followed the same procedures as described above.

Native PAGE folding assay

2 pmol *cis*-ribozyme was allowed to fold in HMN buffer containing 0 – 10 mM $MgCl_2$ following the procedure for the cleavage assay. The RNA was rapidly mixed with 10% glycerol and immediately analyzed by native 8% PAGE in THEM running buffer (66 mM HEPES, 34 mM Tris, 0.1 mM EDTA, and 3 mM $MgCl_2$)(10). The gel was run at 4°C at 15 W for 4 h. The gel was stained with SYBR-Gold and imaged as above.

Template-Based Homology Modeling

To understand the effects of deleterious mutations at the structural level, we modeled the full-length parental (A12G,U31C) *Bsu glmS* ribozyme using the crystal structure of the homologous *Tte glmS* ribozyme (PDB 2H0Z) as a template. All modeling was performed in Molecular Operating Environment (MOE; Chemical Computing Group) using the following strategy: regions that are highly conserved according to (3) were mutated in place to ensure minimal deviation from the crystal structure. To model loops, base pairs or linkers that are absent in the *Tte* sequence, the *Bsu* sequence and surrounding secondary structure (Figure S5) was provided as input into RNAComposer (2). Output structures that were most similar to the *Tte* crystal structure were selected, and the missing segment was excised from the output structure and grafted onto the *Bsu* homology model. Short insertions were constructed using the RNA Builder tool in MOE. A few cycles of energy minimization were carried out on the inserted nucleotides to remove any steric clash or incorrect backbone geometry.

All-Atom Molecular Dynamics Simulations

All-atom molecular dynamics (MD) simulations were performed using the GROMACS 2019.4 software package (11). RNA was represented by the Amber99 force field (12) with Chen-Garcia modifications for RNA bases (13) and Steinbrecher-Case modifications for backbone phosphates (14). Each model was placed in the center of a cubic box such that there was a minimum distance of 1.5 nm from RNA atoms to the box edge in all directions. Models were solvated with TIP4P-Ew, 150 mM KCl and 10 mM $MgCl_2$. $MgCl_2$ was represented using the

Grotz-Schwierz nano-magnesium parameter set for TIP4P-Ew (15). Each system was then energy minimized using the steepest descent algorithm for 10,000 steps with a 2 fs timestep and a force tolerance of 800 kJ $mol^{-1}nm^{-1}$. After energy minimization, all systems were simulated in the NPT ensemble using a leapfrog Verlet integrator with a 2 fs timestep. An isotropic Berendsen barostat was used for pressure coupling (16) at 1 bar with a coupling coefficient of τ_P = 1 ps. A velocity-rescale Berendsen thermostat was used for temperature coupling (17) with a time constant of τ_t = 0.1 ps. The LINCS algorithm was used to constrain all bonds, and a cutoff of 1.0 nm was employed for Lennard-Jones and Coulomb interactions using the Verlet scheme. All simulations were performed at a temperature of 310K. Flat-bottomed distance restraints were applied at strengths of 250 kJ/mol/nm^2 to select contacts during equilibration and production runs to reinforce the secondary structure and global fold. The energetic penalty was only applied if the distance between a pair of atoms fell outside the flat-bottomed range of 1.6Å-4Å.

The *Bsu* homology model was equilibrated at for a total of 37 ns with flat-bottomed distance restraints applied to conserved core contacts, capping base pairs, tertiary contacts between the core and IL4, and the G12-C31-U126 A-minor interaction (Table S7). To confirm the model was sufficiently equilibrated, we used a dimensionless nucleic acid-specific structure deviation metric, eRMSD (18), to compare snapshots at 20 ns and 35 ns to the remaining frames in the trajectory. eRMSD relative to the 20 ns snapshot oscillated around an average of 0.76, and the eRMSD relative to the 35 ns snapshot oscillated around an average of 0.65. Given that an eRMSD value below 0.8 corresponds to structures significantly close to the reference state (18), any snapshot chosen beyond the 20 ns timepoint would have the same structural properties and would thus yield the same results.

The A40G and A114C mutations were introduced into the optimized WT model in MOE. The WT, A114C and A40G models were simulated at 310 K for roughly 50 ns across 10 replicas, for a total of 500 ns of sampling per model (Table S5). Starting conformations were identical, but starting velocities were randomized such that each trajectory evolved differently. To preserve the integrity of the secondary structure during adaptation to the introduced mutations, distance restraints were applied to a subset of core contacts, capping base pairs and the G12-C31-U126 GNRA tetraloop-receptor interaction (Table S7). Hydrogen bonds were analyzed using the MDAnalysis package (19). Base stacking interactions were analyzed using the Dissecting the Secondary Structure of RNA (DSSR) package (20) and an in-house Python script.

SUPPLEMENTAL REFERENCES

1. Klein, D.J. and Ferre-D'Amare, A.R. (2006) Structural basis of glmS ribozyme activation by glucosamine-6-phosphate. *Science*, **313**, 1752-1756.
2. Antczak, M., Popenda, M., Zok, T., Sarzynska, J., Ratajczak, T., Tomczyk, K., Adamiak, R.W. and Szachniuk, M. (2016) New functionality of RNAComposer: an application to shape the axis of miR160 precursor structure. *Acta Biochim Pol*, **63**, 737-744.
3. McCown, P.J., Winkler, W.C. and Breaker, R.R. (2012) Mechanism and distribution of glmS ribozymes. *Methods Mol Biol*, **848**, 113-129.
4. Winkler, W.C., Nahvi, A., Roth, A., Collins, J.A. and Breaker, R.R. (2004) Control of gene expression by a natural metabolite-responsive ribozyme. *Nature*, **428**, 281-286.
5. Wrenbeck, E.E., Klesmith, J.R., Stapleton, J.A., Adeniran, A., Tyo, K.E. and Whitehead, T.A. (2016) Plasmid-based one-pot saturation mutagenesis. *Nat Methods*, **13**, 928-930.
6. Lau, M.W.L. and Ferré-D'Amaré, A.R. (2016) In vitro evolution of coenzyme-independent variants from the glmS ribozyme structural scaffold. *Methods*, **106**, 76-81.
7. Bingaman, J.L., Zhang, S., Stevens, D.R., Yennawar, N.H., Hammes-Schiffer, S. and Bevilacqua, P.C. (2017) The GlcN6P cofactor plays multiple catalytic roles in the glmS ribozyme. *Nat. Chem. Biol.*, **13**, 439-445.
8. Kowalsky, C.A., Klesmith, J.R., Stapleton, J.A., Kelly, V., Reichkitzer, N. and Whitehead, T.A. (2015) High-resolution sequence-function mapping of full-length proteins. *PLoS One*, **10**, e0118193.
9. Virtanen, P., Gommers, R., Oliphant, T.E., Haberland, M., Reddy, T., Cournapeau, D., Burovski, E., Peterson, P., Weckesser, W., Bright, J. *et al.* (2020) SciPy 1.0: fundamental algorithms for scientific computing in Python. *Nat Methods*, **17**, 261-272.
10. Woodson, S.A. and Koculi, E. (2009) Analysis of RNA folding by native polyacrylamide gel electrophoresis. *Methods Enzymol.*, **469**, 189-208.
11. Abraham, M.J., Murtola, T., Schulz, R., Páll, S., Smith, J.C., Hess, B. and Lindahl, E. (2015) GROMACS: High performance molecular simulations through multi-level parallelism from laptops to supercomputers. *SoftwareX*, **1-2**, 19-25.
12. Wang, J., Cieplak, P. and Kollman, P.A. (2000) How well does a restrained electrostatic potential (RESP) model perform in calculating conformational energies of organic and biological molecules? *Journal of Computational Chemistry*, **21**, 1049-1074.
13. Chen, A.A. and García, A.E. (2013) High-resolution reversible folding of hyperstable RNA tetraloops using molecular dynamics simulations. *Proc Natl Acad Sci U S A*, **110**, 16820-16825.
14. Steinbrecher, T., Latzer, J. and Case, D.A. (2012) Revised AMBER parameters for bioorganic phosphates. *J Chem Theory Comput*, **8**, 4405-4412.
15. Grotz, K.K. and Schwierz, N. (2022) Optimized Magnesium Force Field Parameters for Biomolecular Simulations with Accurate Solvation, Ion-Binding, and Water-Exchange Properties in SPC/E, TIP3P-fb, TIP4P/2005, TIP4P-Ew, and TIP4P-D. *J Chem Theory Comput*, **18**, 526-537.
16. Berendsen, H.J.C., Postma, J.P.M., van Gunsteren, W.F., DiNola, A. and Haak, J.R. (1984) Molecular dynamics with coupling to an external bath. *The Journal of Chemical Physics*, **81**, 3684-3690.

17. Bussi, G., Donadio, D. and Parrinello, M. (2007) Canonical sampling through velocity rescaling. *J Chem Phys*, **126**, 014101.
18. Bottaro, S., Di Palma, F. and Bussi, G. (2014) The role of nucleobase interactions in RNA structure and dynamics. *Nucleic Acids Res*, **42**, 13306-13314.
19. Michaud-Agrawal, N., Denning, E.J., Woolf, T.B. and Beckstein, O. (2011) MDAnalysis: a toolkit for the analysis of molecular dynamics simulations. *J Comput Chem*, **32**, 2319-2327.
20. Lu, X.J., Bussemaker, H.J. and Olson, W.K. (2015) DSSR: an integrated software tool for dissecting the spatial structure of RNA. *Nucleic Acids Res*, **43**, e142.
