# Supplementary material for: Optimized periphery-core interface increases fitness of the Bacillus subtilis glmS ribozyme
Source: Nucleic Acids Res. 2024 Sep 25;52(21):13340–50. doi: 10.1093/nar/gkae830 (PMC11602151; doi:10.1093/nar/gkae830)
Supplement: gkae830_Supplemental_Files [file gkae830_supplemental_files.zip › Yu_SIMovieCaptions.docx]

Supplemental Movie S1. Representative trajectory (replica 5) of the WT *Bsu* ribozyme core (cyan) and IL4 (wheat), highlighting interactions of residue A40 (blue). As shown in Figure 6B, A40 helps position the catalytic residues A-1 and G1.

Supplemental Movie S2. Representative trajectory (replica 8) of the A40G *Bsu* ribozyme core (cyan) and IL4 (wheat). As shown in Figure 6C, G40 N2 (blue) forms a non-native hydrogen bond with A141 N3 in IL4. This results in a loss of hydrogen bonds between the catalytic residue G1 and core residues G67, G40 and C54. The conserved base triple involved in cofactor recognition is also disrupted.

Supplemental Movie S3. Representative trajectory (replica 10) of the WT *Bsu* ribozyme core (cyan) and IL4 (wheat) highlighting the contacts between the core and IL4 residue A114. As shown in Figure 7A, A114 (magenta) contacts core residues G55 and G39 to help stabilize the native conformation of the core.

Supplemental Movie S4. Trajectory (replica 9) showing core-IL4 misdocking in the A114C *Bsu* ribozyme. As shown in Figure 7D, C114 (magenta) forms a non-native hydrogen bond with G55 that disrupts the base triple involved in cofactor recognition.
